# Polyamines in the life of Arabidopsis: profiling the expression of *S*-adenosylmethionine decarboxylase (*SAMDC*) gene family during its life cycle

**DOI:** 10.1186/s12870-017-1208-y

**Published:** 2017-12-28

**Authors:** Rajtilak Majumdar, Lin Shao, Swathi A. Turlapati, Subhash C. Minocha

**Affiliations:** 1 Department of Biological Sciences, University of New Hampshire, Durham, NH USA; 2 USDA-ARS, SRRC, 1100 Robert E. Lee Blvd, New Orleans, LA 70124 USA

**Keywords:** Arabidopsis, SAMDC, Promoter, 5’UTR, 3’UTR, Polyamines, Metabolism, Gene family evolution

## Abstract

**Background:**

Arabidopsis has 5 paralogs of the *S*-adenosylmethionine decarboxylase (*SAMDC*) gene. Neither their specific role in development nor the role of positive/purifying selection in genetic divergence of this gene family is known. While some data are available on organ-specific expression of *AtSAMDC*1, *AtSAMDC*2, *AtSAMDC*3 and *AtSAMDC*4, not much is known about their promoters including *AtSAMDC*5, which is believed to be non-functional.

**Results:**

(1) Phylogenetic analysis of the five *AtSAMDC* genes shows similar divergence pattern for promoters and coding sequences (CDSs), whereas, genetic divergence of 5’UTRs and 3’UTRs was independent of the promoters and CDSs; (2) while *AtSAMDC*1 and *AtSAMDC*4 promoters exhibit high activity (constitutive in the former), promoter activities of *AtSAMDC*2, *AtSAMDC*3 and *AtSAMDC*5 are moderate to low in seedlings (depending upon translational or transcriptional fusions), and are localized mainly in the vascular tissues and reproductive organs in mature plants; (3) based on promoter activity, it appears that *AtSAMDC*5 is both transcriptionally and translationally active, but based on it’s coding sequence it seems to produce a non-functional protein; (4) though 5’-UTR based regulation of *AtSAMDC* expression through upstream open reading frames (uORFs) in the 5’UTR is well known, no such uORFs are present in *AtSAMDC*4 and *AtSAMDC*5; (5) the promoter regions of all five *AtSAMDC* genes contain common stress-responsive elements and hormone-responsive elements; (6) at the organ level, the activity of *At*SAMDC enzyme does not correlate with the expression of specific *AtSAMDC* genes or with the contents of spermidine and spermine.

**Conclusions:**

Differential roles of positive/purifying selection were observed in genetic divergence of the *AtSAMDC* gene family. All tissues express one or more *AtSAMDC* gene with significant redundancy, and concurrently, there is cell/tissue-specificity of gene expression, particularly in mature organs. This study provides valuable information about *AtSAMDC* promoters, which could be useful in future manipulation of crop plants for nutritive purposes, stress tolerance or bioenergy needs. The *AtSAMDC*1 core promoter might serve the need of a strong constitutive promoter, and its high expression in the gametophytic cells could be exploited, where strong male/female gametophyte-specific expression is desired; e.g. in transgenic modification of crop varieties.

**Electronic supplementary material:**

The online version of this article (10.1186/s12870-017-1208-y) contains supplementary material, which is available to authorized users.

## Background

**Fig. 1:**
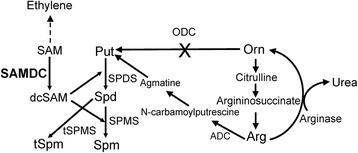
Polyamine biosynthetic pathway. Pathway for the biosynthesis of polyamines in *Arabidopsis thaliana*; abbreviations: ODC, ornithine decarboxylase (absent in Arabidopsis); ADC, Arginine decarboxylase; SAMDC, S-adenosylmethionine decarboxylase; SPDS, spermidine synthase; SPMS, spermine synthase; tSPMS, thermospermine synthase

Gene families consisting of multiple members, which code for the same enzyme are often found in plants and other eukaryotic organisms. A major question in such cases is, whether or not there is an evolutionary advantage of this redundancy to the organism (e.g. higher demand of the protein product - i.e. the importance of the enzymatic function) or the need of functional diversity and differential distribution of the product or both. *S*-adenosylmethionine decarboxylase (SAMDC, *a.k.a.* AdoMetDC - EC 4.1.1.50), a key enzyme for the biosynthesis of higher polyamines (PAs), is encoded by a multigene family in most angiosperms [1–5]. The enzyme carries out a vital rate-limiting step of the biosynthesis of dcSAM from SAM [6, 7], which serves as donor of the aminopropyl moiety for the biosynthesis of two ubiquitous PAs namely spermidine (Spd) and spermine (Spm) by the enzymes Spd synthase and Spm synthase, respectively (Fig. 1). No other major function of dcSAM is known. *Arabidopsis thaliana* apparently has five *SAMDC* genes (Table 1), which show a high degree of sequence similarity amongst them (Additional file 1: Table S1). Two paralogs (*AtSAMDC*1 and *AtSAMDC*2) have been well characterized with respect to their transcription as well as the regulation of their translation via 5’un-translated regions (5’UTRs). In each case, the 5’UTR contains within it two upstream open reading frames (uORFs), which are translated in a PA-dependent manner, and they (or their products) control *SAMDC* mRNA translation [1, 8, 9]. No information is currently available on the translational control of *AtSAMDC*3 (that has only one uORF), and *AtSAMDC*4 and *AtSAMDC*5 (neither of them has a uORF). Even less is known about the genetic divergence of the promoter sequences of the five *AtSAMDC* gene family members in relation to their upstream promoter elements, and the 5’UTRs of the transcript. Likewise, developmental and tissue specific expression of the five *AtSAMDC*s and their role in the regulation of Spd/Spm biosynthesis in Arabidopsis by their corresponding promoters is not well understood.

**Table 1:** Details of the SAMDC genes annotated in the Arabidopsis thaliana database (www.arabidopsis.org). For additional information on AtSAMDC5 gene sequence see NCBI database (https://www.ncbi.nlm.nih.gov/gene/?term=At3g17715)

Gene (Accession no.)	ESTs	5’UTR	ORF (bp)	3’UTR (bp)
*AtSAMDC1* (At3g02470)	1247	1–1106 ORFs: 691–702, 702–865	1107–2207 (1101)	2208–2445 (238)
*AtSAMDC2* (At5g15950)	83	1–837 ORFs: 553–564, 564–719	838–1926 (1089)	1927–2119 (193)
*AtSAMDC3* (At3g25570)	6	1–876 ORFs: 468–635	877–1926 (1050)	1927–2106 (180)
*AtSAMDC4* (At5g18930)	1	1–108	109–1152 (1044)	1153–1599 (447)
*AtSAMDC5* (At3g17715)	6	1–12	13–1176 (1164)	1177–1228 (52)

Abbreviations used: EST, expressed sequence tags; UTR, un-translated region; ORF, open reading frame

Transcripts of *SAMDC* have been characterized in a wide variety of plant species, often with higher expression in reproductive vs. the vegetative organs [1, 4, 10–16]. Expression of *SAMDC* and the role of higher PAs in somatic embryogenesis in carrot (*Daucus carota*) suspension cultures revealed that expression was greater at early stages of embryo development vs. the later stages [10]. In carnation (*Dianthus caryophyllus*), a promoter::5’UTR(*SAMDC*)::*GUS* construct was used to show high activity of GUS in stamens, pollen, stigma and petals with moderate activity in the stem and the cotyledonary veins of young tobacco seedlings [12]. Similar results were reported in apple [*Malus sylvestris* (L.) Mill. var. *domestica*] using RNA gel blot analysis [4]. In Arabidopsis, the transcripts of *AtSAMDC*1, *AtSAMDC*2, *AtSAMDC*3 and *AtSAMDC*4 were detected (using northern blots, RT-PCR and micro-arrays) in vegetative as well as in the reproductive organs [1, 11, 14, 17]**.** Differential distribution of *AtSAMDC* RNAs showed that (i) *AtSAMDC*1 was expressed in all organs of mature plants; (ii) the expression of *AtSAMDC*2 was high in roots, leaves and flowers; (iii) *AtSAMDC*3 showed weaker expression in all organs except for the siliques; (iv) the expression of *AtSAMDC*4 was low but ubiquitous; however, for *AtSAMDC*5, not much has been reported about its expression pattern during the life of the plant.

Several attempts involving transgenic expression of homologous or heterologous *SAMDC* genes in a wide variety of plant species have often resulted in 2- to 4-fold higher contents of Spd and Spm with or without effects on the phenotype [18–26]. Metabolic consequences of *SAMDC* over-expression may affect pathways beyond its direct target of PA biosynthesis as was seen for the genetic manipulation of putrescine (Put) in poplar (*Populus nigra x maximowiczii*) and Arabidopsis [27–29]. In tomato using a fruit specific *E8* promoter with yeast *SAMDC*, a greater accumulation of metabolites, such as Gln, Asn, choline, citrate, fumarate, malate, and changes in fatty acids, etc. were observed (besides increase in Spd and Spm) in transgenic fruits ripened off the vines as compared to their WT counterparts [26, 30].

Numerous studies have reported up-regulation of *SAMDC* in plants in response to a wide variety of stresses including salt, drought, high temperature, low temperature, oxidative stress, etc.; in line with the suggested correlations between PAs and abiotic stresses [4, 11, 17, 25, 31–37]. However, these studies are limited in the number of developmental stages that were analyzed (except those using micro-arrays), and/or in localizing the activity of specific paralogs in cell/tissue types [4, 11, 17]. Specifically, no data are currently available on the expression and the role of *AtSAMDC*5, which is believed to be transcriptionally inactive [1].

Since *AtSAMDC* genes often have highly complex 5’UTRs, highly conserved coding sequences, produce an enzyme with extremely short half-life (often <20 min), and show a divergence into a five-member family in *A. thaliana*, a major question of interest would be if positive selection played a major role in genetic divergence of this gene family with a rather conserved single enzymatic function. Given the importance of PAs in the life of plants, and a high degree of sequence similarity among the five *AtSAMDC* paralogs, the present study was undertaken to comprehensively characterize the expression pattern (at cell/tissue to organ level) of all five members of the *AtSAMDC* gene family during the life cycle of *A. thaliana* using promoter::*GUS* fusion approach. These data were then verified with QRT-PCR of transcripts of the five genes in selected organs and developmental stages. To complete the story of *AtSAMDC* expression, enzyme activities as well as PA contents in various organs were also analyzed at different stages of development. While it would have been interesting, the specific contribution of the enzyme/protein in specific cell (or even tissue/organ) types was not done, because the individual proteins or enzyme activities cannot be easily distinguished from each other in a common cellular environment. Finally, the putative promoter regions of all *AtSAMDC* paralogues were subjected to bioinformatics analyses for comparison of the key *cis-*regulatory elements within them. The results reveal different selection criteria for genetic divergence among different members of the *AtSAMDC* gene family, and show distinct differences in expression patterns of the five genes in different tissues/organs of the plant during development.

## Methods

### Phylogenetic trees

Evolutionary analyses of genomic sequences were conducted in MEGA version 5.2.2 [38] using MUSCLE software [http://www.drive5.com/muscle/, [39]. The phylogenetic relationships among the *AtSAMDC* upstream elements, CDSs and respective 5′- and 3’-UTRs were inferred by using the Maximum Likelihood method based on the Tamura-Nei model [40] with boot strapping. The tree with the highest log likelihood was chosen and initial tree(s) for the heuristic search were obtained automatically by applying Neighbor-Join and BioNJ algorithms to a matrix of pairwise distances estimated using the Maximum Composite Likelihood (MCL) approach, and then selecting the topology with superior log likelihood value. Boot strapping with 500 replications was conducted as a test of phylogeny and the trees were visualized using Fig tree software version 1.4.0 (http://tree.bio.ed.ac.uk/software/figtree/).

### Analysis of promoters and UTRs

The upstream (up to 700 bp) promoter regions of the five *AtSAMDC* genes were compared using GATA program [41]. While the distance between a specific *SAMDC* gene and its 5′ upstream neighboring gene varied from ~700 bp to ~4 kb, the 700 bp upstream regions (somewhat arbitrarily) of the putative promoter sequences were taken into consideration for GATA analysis among different pairs. The sequences were aligned and plotted by GATA aligner and plotter with a window size of 7 and lower cut off score of 12 bit for graphically visualizing the inversions and duplications within the compared pair of sequences. Promoter Wise 2 package of EMBL-EBI [http://www.ebi.ac.uk/Tools/psa/promoterwise/] was used to predict alignments in reverse and similar orientation among the pairwise sequence comparisons; the putative promoter sequences were aligned using ClustalW2 [42] - http://www.ebi.ac.uk/Tools/msa/clustalw2/]. For non coding sequences such as promoters and UTRs, Tajima test of neutrality was conducted in MEGA version 7 to determine the type of selection [43]. The total number of segregating sites among all promoter sequences were estimated along with nucleotide diversity per site. Similar analyses were performed with 5′ and 3’ UTR sequences also. Since there are no codons present, the analyses were conducted by choosing nucleotide as a substitution type and the Tajima test D-value was recorded for each dataset. This test was conducted to find out whether the evolution process followed a positive (balancing selection or a non-random process) or a negative (purifying or a random process) selection. Significant positive selection is indicated by a higher positive Tajima D-value (>2), whereas a higher negative value (> −2) implies a purifying selection with greater confidence levels [43].

### Analysis of coding sequences

For *SAMDC* coding sequences, the ratios of the rate of non-synonymous (*Ka*) to synonymous (*Ks*) substitutions were estimated in K-Estimator program by following Yang and Neilsen method [44, 45].

### Analysis of *cis* elements in the *SAMDC* promoters

For analysis of putative *cis* elements in the promoter regions, Athena promoter analysis tool was used [46]. The loci IDs of different *AtSAMDC*s were used to identify putative *cis* elements in the promoter regions.

### Generation of the promoter::*GUS* fusion transgenic plants

Respective genomic fragments of *AtSAMDC* constructs (summarized in Fig. 3) were PCR amplified from *A. thaliana* (Col-0) genomic DNA using sequence specific primers (Additional file 1: Table S2) and cloned into pCR8.0/GW/TOPO entry vector (Invitrogen, Carlsbad, CA). The fragments in the entry vector were subsequently recombined into the Gateway-compatible pMDC163 plant destination vector [47] containing *GUS* reporter gene using LR Clonase reaction kit (Invitrogen). The plasmids were used to transform *Agrobacterium tumefaciens* (strain GV3101) using electroporation. For *AtSAMDC*1 (*SAMDC*-1A) and *AtSAMDC*2 (*SAMDC*-2A, 2B) constructs, genomic fragments (Fig. 3) were initially cloned into pCR2.1/TOPO vector, and then recombined into the plant destination vector pCAMBIA1381 containing the *GUS* gene.

*Arabidopsis thaliana* plants were transformed with *A. tumefaciens* containing the recombinant plasmid by floral dip method [48]. Five independent T_2_ transgenic lines were selected that had a single insertion of the cloned cassette (tested by segregation analysis on hygromycin). The progenies of the lines following 3:1 segregation pattern for hygromycin tolerance were considered having single copy transgene insertion, and showed GUS activity at each generation. These lines were grown to obtain T_3_ generation seeds. Screened independent homozygous T_3_ or higher generation lines were used for all experiments.

### Growth conditions and treatments

Arabidopsis seedlings were grown at 25 ± 1 °C under 16 h photoperiod (80 ± 10 μE m^−2^ s^−1^) on solid germination medium (GM) [29]. For qualitative analyses of GUS at mature stage, plants were grown in soil mix containing 3 parts Scott’s 360 Metro-Mix (Scotts Company, Marysville, OH) and 1 part perlite in 3″square pots. The growth conditions were 21 °C under 18 h photoperiod (80 ± 10 μE m^−2^ s^−1^). Plants were watered on alternate days from below and fertilized with addition of ¼ suggested strength of Miracle-Gro**®** (Scotts Company) synthetic fertilizer applied with irrigation water every 5th day.

### Transient promoter activity in poplar

The biolistic bombardment protocol for transient GUS activity was performed in poplar (*Populus nigra x maximowiczii*) cells [49]. The plasmid DNA (normalized to ~1.5 μg DNA/mg gold particles) was coated onto 1.5–3 μm gold particles (Aldrich, Milwaukee, WI) in the presence of 1.0 M CaCl_2_ and 16.7 mM Spd, and bombarded by Bio-Rad PDS 1000/He Biolistic Bombardment Apparatus (*a.k.a.* the gene gun) (Hercules, CA), with either 1100- or 1350-psi rupture discs. Approximately 48 h after bombardment, the cells were treated with GUS reaction mix containing 5-bromo-4-chloro-3-indonyl-β-D-glucuronide (X-Gluc) in 1.0 mM potassium ferricyanide, 1.0 mM potassium ferrocyanide, 100 mM sodium phosphate buffer pH 7.0, 5 mM EDTA, 0.1% Triton X-100, and 20% methanol [50], for 18–20 h at 37°C, and numbers of blue cells were counted under a dissecting microscope.

### **Visualization of** β**-Glucuronidase (GUS) activity in plants during development**

Histochemical activity of GUS was visualized by submerging *A. thaliana* plants collected at different developmental stages in the GUS reaction mix described above and vacuum infiltrated for 5 min. Following incubation at 37 °C for 18–24 h, the reaction mix was removed and the samples were kept in 70% ethanol (for removal of chlorophyll background) at 4 °C until analysis [51]. Representative photographs were taken using an Olympus C650 digital camera mounted on an Olympus SZX9 dissecting microscope.

### RNA isolation, cDNA synthesis and QRT-PCR

Plant samples stored at −80 °C were used for total RNA extraction using the ZR Plant RNA MiniPrep™ Kit (Zymo Research, Irvine, CA) using RQ1 RNase-Free DNAse (Promega, Madison, WI); RNA was quantified using Nano-Drop spectrometer (Thermo-Fisher Scientific, Madison, WI). RNA samples were reverse transcribed to first strand cDNA using qScript™ cDNA SuperMix kit using manufacturer’s guidelines (Quanta Biosciences, Gaithersburg, MD). The reaction conditions were 5 min at 25 °C, 30 min at 42 °C and 5 min at 85 °C. The resultant cDNA was stored at −20 °C before QRT-PCR analysis.

Gene expression was quantified by SYBR-green dye based assay in a 10 μl reaction containing 1× final concentration of Low ROX SYBR-Green FastMix (Quanta Biosciences) with a final concentration of 50 nmol each of the forward and the reverse sequence-specific primers (Additional file 1: Table S2), and template cDNA. The reactions were run in MicroAmp™ Fast Optical 96-Well Reaction Plate in AB7500 Fast Real-Time thermocycler (Applied Biosystems, Foster City, CA). The thermocycler conditions included a pre-incubation at 50 °C for 2 min, dye activation at 96 °C for 15 s, primer annealing at 55 °C for 30 s, and extension at 72 °C for 1 min. A dissociation curve around 60 °C to 95 °C confirmed that the signal was due to the interaction between SYBR-green and the gene specific amplicon. A standard curve was prepared from a series of 2-fold serial dilutions over a range of 20-fold of cDNA. The value of gene expression and the reference gene expression for a specific sample was extrapolated from standard curves obtained from gene specific primers and reference gene primers. All gene expression data were normalized to the *AtTIP-*41 (At4g34270) as reference gene [52, 53].

### SAMDC enzyme assay and quantification of polyamines

The activity of SAMDC was measured [54] using 0.1 μCi of (1-^14^C)–SAM [specific activity 58 mCi mmol^−1^; Moravek, Ca]. The reaction was run for 60 min at 37 °C. Enzyme activity is expressed as nmol CO_2_.h^−1^.mg^−1^ soluble protein. Total soluble protein contents were analyzed in tissue extracts in potassium phosphate buffer (0.1 M; pH 7.0) by the Bradford [55] method using bovine serum albumin as standard.

Arabidopsis wild type (WT) plants were grown in solid GM (for seedlings) and in pots (as described above) and collected in 9× volume of 5% HClO_4_ (*v*/v ~0.77 N). The samples were frozen and thawed (at room temperature) for three times before quantification of PAs by HPLC [56]. Our HPLC analysis did not distinguish between Spm and thermospermine.

## Results

### Phylogenetic analysis

**Fig. 2:**
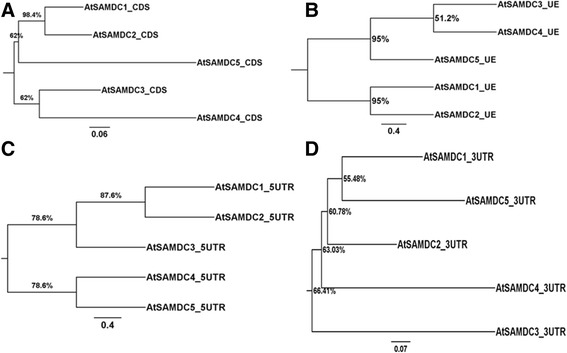
Phylogenetic analyses of the *SAMDC* genes in *Arabidopsis thaliana*. **a** CDS; **b** upstream elements (UE: promoter); **c** 5’UTRs; and **d** 3’UTRs. Evolutionary analyses of the *SAMDC* sequences was inferred by using the Maximum Likelihood method based on the Tamura-Nei model with boot strapping. The tree with the highest log likelihood is presented here for each phylogenetic analysis. Initial tree(s) for the heuristic search were obtained automatically by applying Neighbor-Join and BioNJ algorithms to a matrix of pairwise distances estimated using the Maximum Composite Likelihood (MCL) approach, and then selecting the topology with superior log likelihood value. Boot strapping with 500 replications was conducted as a test of phylogeny. The percentage of trees in which the associated taxa clustered together is shown next to the branches defined as percent boot strap values. The tree was drawn to scale, with branch lengths measured as number of nucleotide substitutions per site given in the scale bar

Phylogenetic analysis of the coding sequences of five *AtSAMDC*s showed that *AtSAMDC*1 and *AtSAMDC*2 are grouped close to each other on one node with highest (>98%) boot strap values (Fig. 2A), and *AtSAMDC*3 and *AtSAMDC*4 are grouped closer on another node with *AtSAMDC5* almost equally close to both groups. The phylogenetic analysis of the putative promoter elements also exhibited similar pattern (Fig. 2B). The 5’UTRs on the other hand showed a different pattern compared to CDSs and promoters; whereas *AtSAMDC*1 and *AtSAMDC*2 are found to be closer on one node of the tree and 5’UTRs of *AtSAMDC*4 and *AtSAMDC*5 are present on one branch, the 5’UTR of *AtSAMDC3* showed divergence from both of these two pairs (Fig. 2C). In contrast, the 3’UTRs followed still another pattern in that the *AtSAMDC*1 and *AtSAMDC*5 3’UTRs were on one branch, and *AtSAMDC*2, *AtSAMDC*3 and *AtSAMDC*4 were on separate nodes (Fig. 2D). None of these trees show polytomy and this shows the divergence could be predictable.

When comparisons of different regions of the genes were extended to other species, it was found that among the different *SAMDC* CDSs, *AtSAMDC*1, *AtSAMDC*2 and two *BjSAMDC*s shared higher homology, whereas *AtSAMDC*3, *AtSAMDC*4, and *AtSAMDC*5 were distantly related (Additional file 1: Fig. S1); this indicates more divergence among the five different *AtSAMDCs* than between *SAMDC*s of other taxonomically unrelated species. The details of genomic structures of *AtSAMDC* orthologs are shown in Additional file 1: Table S3. The same phylogenetic pattern was also observed in a comparison for the 5’UTRs of the *SAMDC*s, where *AtSAMDC1* and *AtSAMDC*2 5’UTRs were closer to the 5’UTRs of *BjSAMDC*s (Additional file 1: Fig. S2), but the *AtSAMDC*4 5’UTR was on the same branch as the human *SAMDC* (*HsSAMDC*) 5’UTR.

### Comparison of 5’UTRs among different Arabidopsis *SAMDC* genes

Sequence analyses of *AtSAMDC* cDNAs/genes show that *AtSAMDC*1, *AtSAMDC*2 and *AtSAMDC*3 have relatively large 5’UTR sequences ranging from 876 to 1106 bp, whereas *AtSAMDC*4 has a much shorter 5’UTR of 108 bp in length. *AtSAMDC*5 has extremely small 5’UTR of only 12 bp (Table 1). The presence of highly conserved overlapping ‘tiny’ and ‘small’ uORFs, an important characteristic of plant *SAMDC*s [1, 4, 5], is seen in the 5’UTRs of *AtSAMDC*1 and *AtSAMDC*2; in both cases, the last base of stop codon of the tiny uORF is the first base of the start codon of the small uORF [1, 8]. Introns are only observed in the uORFs and are lacking in the main ORF of *AtSAMDC* genes. It is further noted that while the length of the tiny uORFs is 12 bp, (when present), the small uORF varies in length from 156 to 168 bp (Table 1). The location of tiny and small uORFs varies between 553 and 865 bp downstream of the transcription start site in *AtSAMDC*1, *AtSAMDC*2 and *AtSAMDC*3 genes. The *AtSAMDC*3 contains only small uORF in its 5’UTR, whereas *AtSAMDC*4 and *AtSAMDC*5 do not have a uORF in their 5’UTRs.

### Analysis of putative promoter elements, UTRs, and coding regions of *AtSAMDC* genes

Even though there are conserved domains, and there is microsynteny among the upstream regions in the *AtSAMDC* paralogs, there was also divergence within this region as shown by the presence of inversions and gene duplications (Additional file 1:Fig. S3). Tajima test of neutrality conducted on the non coding regions such as promoter sequences, and 5′ and 3’ UTRs of SAMDC gene family suggested positive or balancing selection (Table 2). This test relies heavily on the average nucleotide diversity (π), mean number of pairwise differences among sequences (θ) and the ratio of the number of segregrating sites vs. total number of nucleotide sites among the sequences. Our results showed a higher (>2) positive Tajima D-Value for SAMDC promoter sequences indicating a positive or balancing selection with an excess of high frequency variants (Table 2). Similarly, positive D-values were observed with 5′ and 3’ UTR sequences. However, in UTR sequences, the D-values were <2 (not significant) implying a mix of high frequency and rare variants in these sequences. Among the different gene combinations for which K estimator assessed the rate of substitutions, *AtSAMDC*1 vs. *AtSAMDC*4 and *AtSAMDC*3 vs. *AtSAMDC*5 showed positive selection while other combinations exhibited purifying selection (Table 3).

**Table 2:** Tajima test results for *SAMDC* non-coding upstream and downstream DNA sequences

Type of sequences	Total number of sequences (m)	Total number of sites (N) in the final dataset	Total number of segregating sites (S)	Number of segregating sites/number of nucleotide sites *pS = S/N*	Mean number of pairwise differences among sequences (Θ)	Nucleotide diversity or nucleotide differences per site (π)	Tajima *D*-Value	Inference based on Tajima *D*-Value
*AtSAMDC* promoters	5	394	333	0.845178	0.405685	0.524112	2.229599*	Positive selection
*AtSAMDC* 5’UTRs	5	12	6	0.500000	0.240000	0.283333	1.240997	Positive selection
*AtSAMDC* 3’UTRs	5	43	32	0.744186	0.357209	0.441860	1.774392	Positive selection

Tajima test [43] was conducted in MEGA Version 7 on an aligned sequence dataset by choosing nucleotide in the substitutions type. N is the total number of nucleotide positions considered by test statistic for the analysis. Any nucleotide site that shows two or more nucleotides among the total number of sequences was considered as a segregating site, which is a default setting. The mean number of pairwise differences between the sequences was represented as θ. The nucleotide differences per site among the sequences was represented as π and was collectively termed as nucleotide diversity. A high positive Tajima D-value of (>2) with * indicates a significant (*P* < 0.05) positive selection in these sequences

**Table 3:** Number of synonymous to non-synonymous substitutions among coding sequences of the *SAMDC* genes estimated using K-estimator software by Yang and Nielson method [45]

Sequence compared	Ka	Ks	Ka/Ks	*P*-Value	Type of selection	*S*	*N*	Divergence-Time
*AtSAMDC*1vs2	2.24	2.50	0.89	0.73	Purifying	156	468	2.30
*AtSAMDC*1vs3	2.03	3.94	0.52	0.0001**	Purifying	159	420	2.49
*AtSAMDC*1vs4	4.75	2.06	2.31	0.01**	Positive	158	432	3.95
*AtSAMDC*2vs4	2.89	4.11	0.70	0.72	Purifying	154	452	3.19
*AtSAMDC*3vs5	3.19	1.59	2.01	0.03*	Positive	130	420	2.78

Ka - rate of non-synonymous substitutions; Ks - rate of synonymous substitutions; Ka/Ks - ratio of non-synonymous to synonymous substitutions; S - number of synonymous substitutions; N - number of non-synonymous substitutions. Divergence time per site per year is estimated by Ka/Ks – Calculator, which is a weighted average of synonymous substitution rate and non-synonymous substitution rate. Significance is tested by Fisher exact test; * denotes *P* < 0.05, ** denotes *P* < 0.01. A significant value for positive selection indicates higher rate of non-synonymous substitutions and purifying indicates higher rate of synonymous substitutions

Bioinformatics analysis of the (700 bp segment) cloned putative promoter regions of all five *AtSAMDC* genes revealed the presence of several commonly recognized *cis*-regulatory motifs (transcription factor binding sites) in most of them (Table 4). Details of their specific names, sequences of these motifs, location within the promoter region, and their general regulatory functions are shown in Additional file 1: Tables S4-S8. The promoters of *AtSAMDC*1 and *AtSAMDC2* contained GAREAT, GADOWNAT (GA responsive elements) motifs, and also the CACGTG, MYB1AT and MYB4, and Box II motifs (light responsive element). Presence of several common stress-response motifs; e.g. ABRE-like, cold stress, and MYB1AT (dehydration response) were also found in these promoters. The unique motifs in *AtSAMDC*1 promoter sequence are W box (pathogenic response and wounding), Z box (developmental expression), and MYB binding site (developmental and stress). On the other hand, promoter sequence of *AtSAMDC*2 contained CARGCW8GAT (AGL-15 site, regulating embryogenesis), I box (light response), RAV1-B (DNA-binding protein), MYCATERD1 (early response to dehydration) and AtMYC2 BS in RD22 (dehydration responsive element) motifs. Besides sharing common motifs in the promoter regions associated with abiotic stresses, and developmental regulation among different *SAMDC*s; *AtSAMDC*4 and *AtSAMDC*5 had an auxin binding site factors motif (ARF).

**Table 4:** Common motifs present in the 700 bp upstream promoter regions of the Arabidopsis *SAMDC* genes

Motif name	Consensus sequence	Physiological responses	*SAMDC* gene promoters containing motifs (upstream location)
ABRE-like binding site motif	CACGTGTA and TCCACGTG	dehydration, low temperature	*AtSAMDC*1 (426), *AtSAMDC*2 (260, 262, 451)
ACGTABREMOTIFA2OSEM	ACGTGTC	ABA responsive expression	*AtSAMDC*1 (426), *AtSAMDC*2 (260, 282, 472)
ARF binding site motif	TGTCTC	auxin responsive element	*AtSAMDC*4 (715, 838), *AtSAMDC*5 (526)
BoxII promoter motif	TTAACC	Transcriptional activator	*AtSAMDC*4 (177, 356), *AtSAMDC*5 (377)
CACGTGMOTIF	CACGTG	essential for beta phaseolin gene expression during embryogenesis	*AtSAMDC*1 (428), *AtSAMDC*2 (262, 453)
CCA1 binding site motif	AGATTGTTAGATTGTT	phytochrome regulation	*AtSAMDC*3 (236, 555), *AtSAMDC*5 (180)
GADOWNAT	ACGTGTC	GA down regulated expression during seed germination	*AtSAMDC*1 (426), *AtSAMDC*2 (260, 472)
GAREAT	TAACAAG	GA induced seed germination	*AtSAMDC*1 (276), *AtSAMDC*2 (615, 659), *AtSAMDC*5 (871, 893)
I box promoter motif	CTTATC	light regulated expression	*AtSAMDC*2 (328)
MYB1AT	TGGTTA	dehydration	*AtSAMDC*1 (410, 639), *AtSAMDC*2 (724), *AtSAMDC*3 (659, 710), *AtSAMDC*4 (355, 510, 975)
MYB4 binding site motif	ACCAAAC	drought, salt, cold, wounding	*AtSAMDC*1 (223), *AtSAMDC*2 (681), *AtSAMDC*3 (150, 232, 275, 723), *AtSAMDC*4 (428), *AtSAMDC*5 (487, 593)
MYB1LEPR	AACTAAC	defense related gene expression	*AtSAMDC*4 (428), *AtSAMDC*5 (593)
RAV1-B binding site motif	CACCTG	domain for DNA binding protein	*AtSAMDC*2 (508), *AtSAMDC*4 (418)
RY-repeat promoter motif	CATGCATG CATGCATG	seed protein related	*AtSAMDC*3 (565)
TATA-box Motif	TATAAA	transcription	*AtSAMDC*1 (61, 122, 379), *AtSAMDC*2 (26), *AtSAMDC*3 (403, 479, 550), *AtSAMDC*4 (134, 397, 679), *AtSAMDC*5 (89)
T-box promoter motif	ACTTTG	transcriptional activator	*AtSAMDC*4 (559), *AtSAMDC*5 (548, 697)
W-box promoter motif	AGTCAA	wound response	*AtSAMDC*1 (103, 652), *AtSAMDC*3 (201), *AtSAMDC*4 (523)

Details of other motifs present in 1 kb (where available) of the promoter region are presented in the Additional file 1; Tables S4-S8

### Transient expression of the promoter::*GUS* constructs

**Fig. 3:**
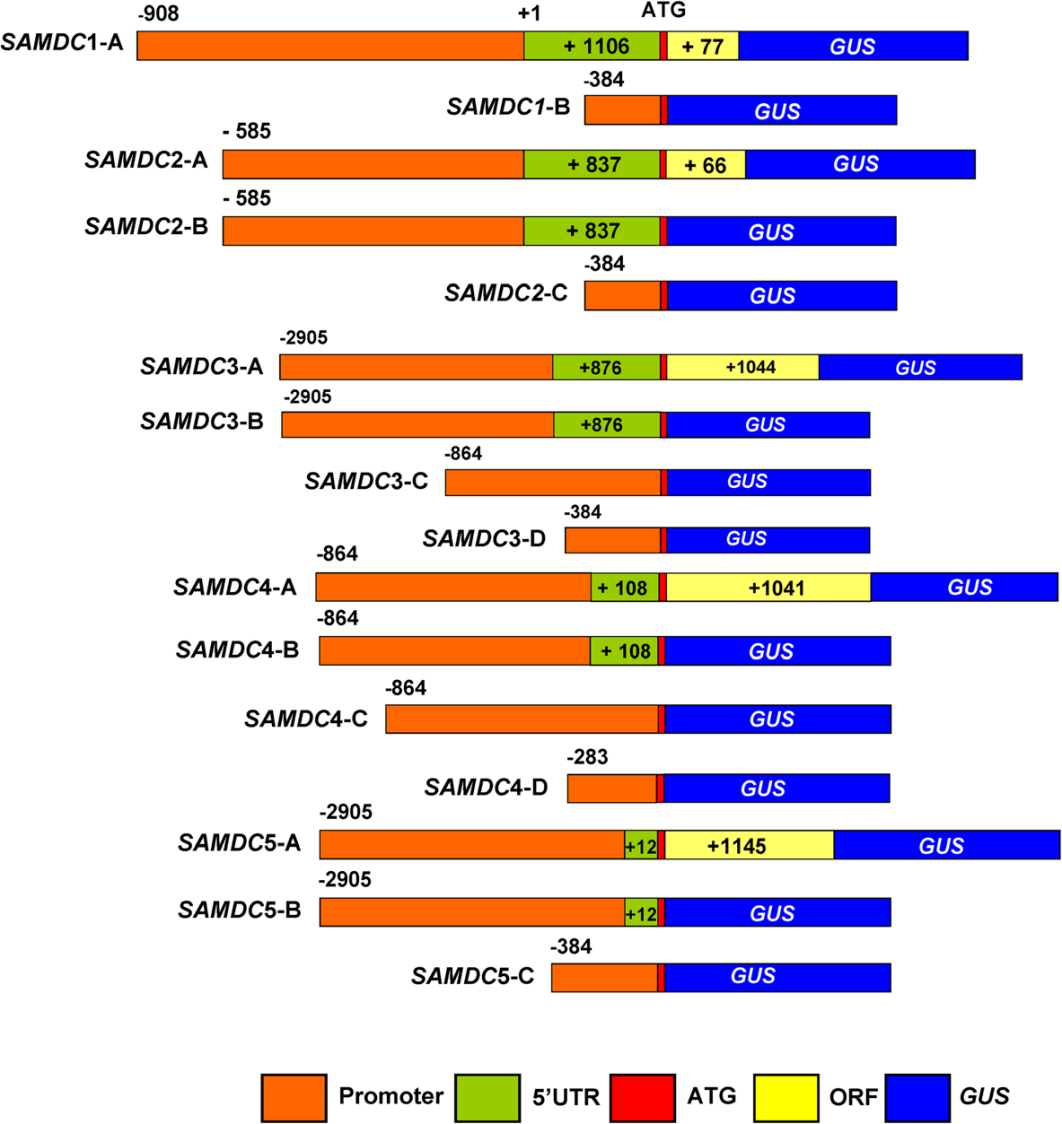
Different constructs of Arabidopsis *SAMDC* genes. *AtSAMDC*1, *AtSAMDC*2, *AtSAMDC*3, *AtSAMDC*4, and *AtSAMDC*5 *GUS* fusions used for expression analyses of the reporter gene (+1 denotes transcription start site)

In order to examine if the various promoter::*GUS* constructs (shown in Fig. 3) were functional in terms of *GUS* activity, a transient expression experiment was performed using biolistic bombardment in a heterologous suspension cell culture system of poplar (*Populus nigra x maximowiczii*) [49]; pCAM-2x*35S*::*GUS* (CaMV2x*35S*::*GUS*) was used for comparison. The data summarized in Additional file 1: Fig. S4 show that: (i) All of the *AtSAMDC*::*GUS* constructs prepared from various putative promoter sequences showed expression in poplar; (ii) none of the promoter::*GUS* combinations were equal to or better than the CaMV2x*35S* promoter; (iii) *AtSAMDC*4-D plasmid, with the shortest putative promoter region of all constructs, showed the highest transient activity of GUS as compared to the other plasmids used in this experiment, with its activity being only slightly less than the control 2x*35S* promoter; (iv) all *AtSAMDC*4::*GUS* constructs had higher activity than the other promoter constructs except *AtSAMDC*1; (v) the activity of GUS was quite different when different segments of the same promoter were used; (vi) *AtSAMDC*5 promoter activity was the lowest of all constructs tested.

Within each promoter, different constructs having/missing different regions of the promoter sequence showed that the presence of the 5’UTR or the *AtSAMDC*-ORF had variable effects in different promoters. For example, *AtSAMDC*4-D with the shortest promoter sequence, showed maximum transient activity of GUS in poplar, and the presence or absence of the 5’UTR and/or the *AtSAMDC*4 ORF did not significantly affect transient expression. The *AtSAMDC*2-A translational fusion had 4-fold higher activity than the translational fusions *SAMDC*3-A and *AtSAMDC*5-A. The absence of the *AtSAMDC* ORF increased GUS activity in both *AtSAMDC*3 and *AtSAMDC*5, and the absence of 5’UTR increased the activity of GUS in *AtSAMDC*3 promoter. The pCAM-*2x35S*::*GUS* construct used as a control, showed the highest expression of all. Although the absolute numbers of blue spots (cells showing GUS activity) varied among plates in different replicate experiments, overall the relative expression of different constructs within a promoter was consistent with GUS activity later seen in the seedlings and the mature plants of Arabidopsis.

### Activity of different promoter constructs at seed germination and early seedling growth

**Fig. 4:**
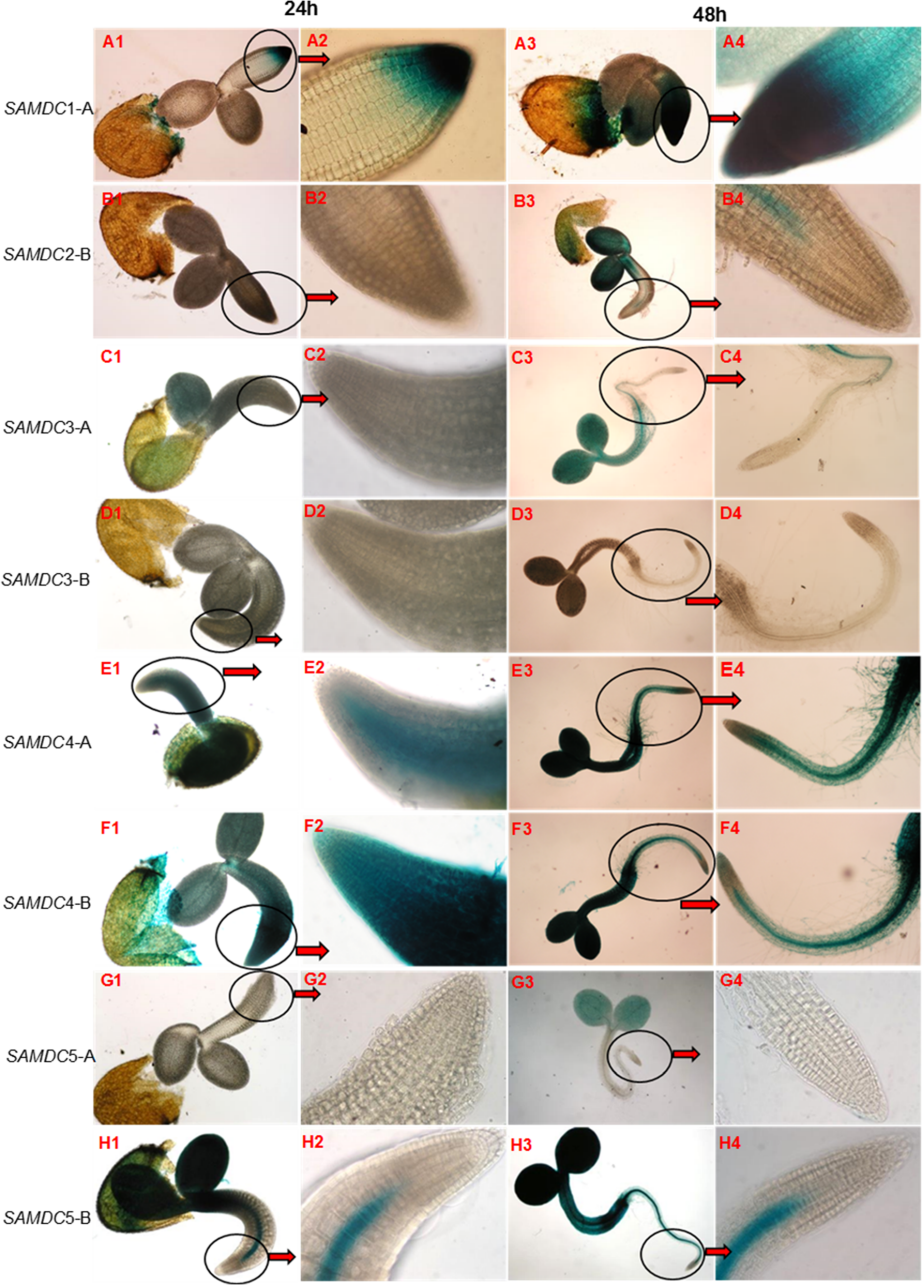
Activity of GUS at early germination stages (24 h and 48 h) in different *AtSAMDC* constructs. *AtSAMDC*1 (A1-A4), *AtSAMDC*2 (B1-B4), *AtSAMDC3* (C1-C4, D1-D4), *AtSAMDC4* (E1-E4, F1-F4) and *AtSAMDC5* (G1-G4, H1-H4) Arabidopsis seeds. The name of individual construct is at the left of individual horizontal panel

Transgenic *A. thaliana* plants of different translational and transcriptional fusion constructs of the five *AtSAMDC* genes (shown in Fig. 3) were used to analyze GUS activity at 24 h and 48 h of incubation in the dark at 4 °C, and later at 1, 3, 7, and 9 days after germination. In order to avoid redundancy of results, the activity of GUS for different *AtSAMDC* constructs in the seedlings presented later is only at 9 days after germination. The transgenic seeds that were tested at 24 h and 48 h while in cold and dark did not have visible germination at that time and were not tested for GUS activity. Germination took place 24–48 h after the plates were transferred to 24 °C growth chamber. Seeds collected at 24 h and 48 h after germination, *AtSAMDC*1-A::*GUS* construct showed high GUS activity at the root tip and some in the seed coat as well; by 48 h, GUS activity was intense and spread throughout the germinating embryo (Fig. 4 A1-A4). For *AtSAMDC*2-B construct, a similar pattern (but relatively lower than *AtSAMDC*1A) of GUS activity was observed except that there was no activity at the root tip (Fig. 4 B1-B4). The *AtSAMDC*2-A construct showed no activity during the 48 h of germination. No GUS activity was detected in the roots for either translational or transcriptional fusions of *AtSAMDC*3 (Fig. 4 C1-C4, D1-D4); the latter showing little activity in the cotyledons as well. For both of *AtSAMDC*4-A and *AtSAMDC*4-B constructs, intense blue color was observed in the seed coat and throughout the seedlings at 24 h; even more so at 48 h (Fig. 4 E1-E4, F1-F4). For *AtSAMDC*5-A, while the presence of the coding region (translational fusion) showed no GUS activity in the roots, at 48 h GUS activity in the cotyledons was seen at 48 h (Fig. 4 G1-G4), However, in the absence of the coding region (i.e. transcriptional fusion), there was strong activity of *GUS* in root vascular system (within 24 h) and the entire shoot region (Fig. 4 H1-H4).

**Fig. 5:**
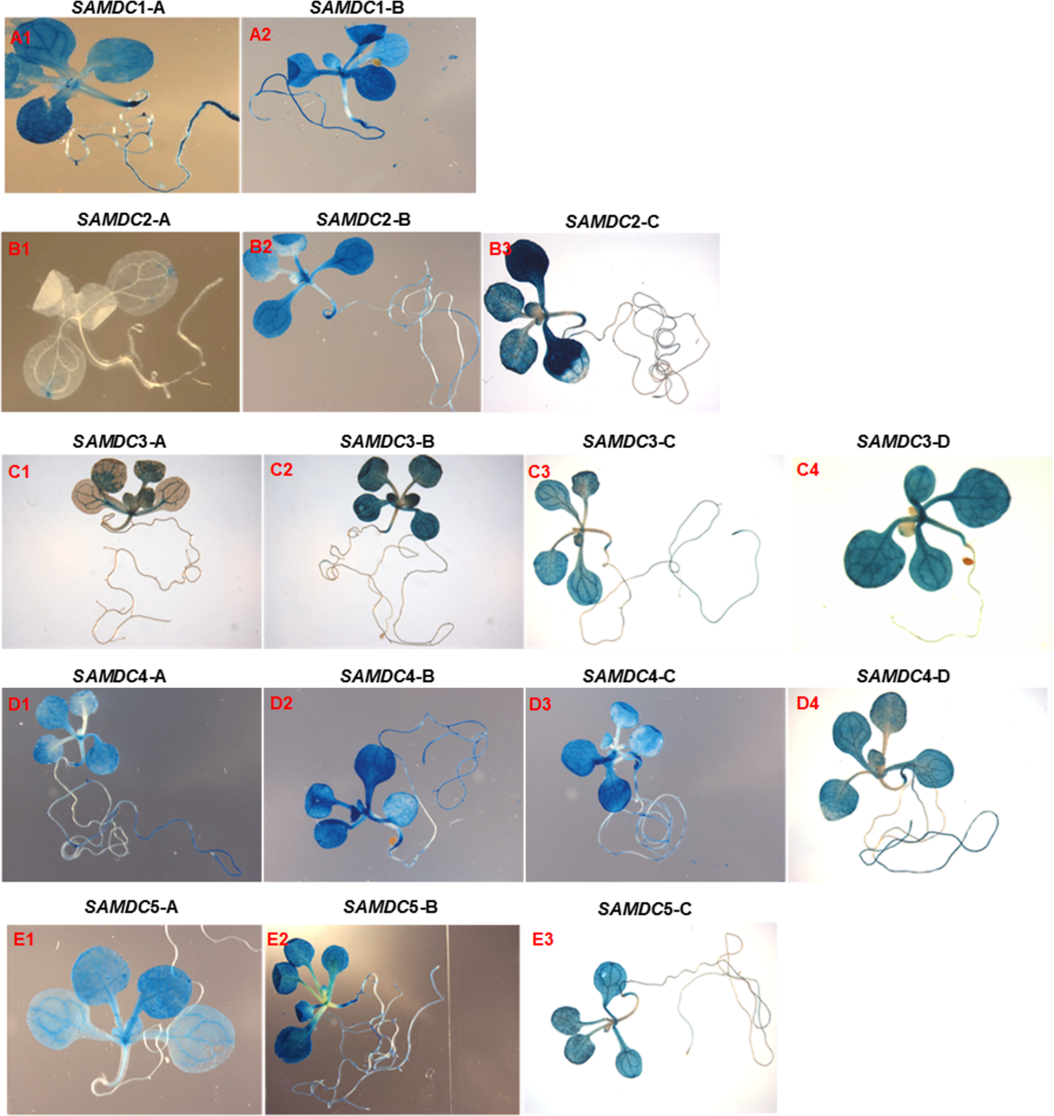
Activity of GUS in 9-day old seedlings. *AtSAMDC*1 (A1-A2), *AtSAMDC*2 (B1-B3), *AtSAMDC*3 (C1-C4), *AtSAMDC*4 (D1-D4), and *AtSAMDC*5 (E1-E3)

More pronounced differences in the activity of GUS in various tissues and organs appeared as the seedlings grew bigger (Fig. 5). Whereas high and uniformly distributed activity of GUS was observed in the *AtSAMDC*1 constructs at day 9, *AtSAMDC*2 showed distinct differences with time, distribution in different tissues/organs, and the effect of part of its 5’UTR + ORF. The promoter without the ORF (i.e. *AtSAMDC*2-B) showed GUS activity ranging from uniform distribution in the cotyledons early on to ubiquitous activity by day 9 (Fig. 5 B2, B3). *AtSAMDC*2-A, which contains 22 codons of the ORF along with the 5’UTR, showed highly restricted GUS activity in the seedlings (Fig. 5 B1). While activity of GUS in the roots for *AtSAMDC*1 was quite high, *AtSAMDC2*-A construct did not show GUS activity in the roots. Removal of 5’UTR significantly increased GUS activity in *AtSAMDC*1-B and *AtSAMDC*2-C constructs even with a promoter of 384 bp in length (Fig. 5 A2, B3).

For *AtSAMDC*3 also, presence of the ORF + 5’UTR (*AtSAMDC*3-A construct) exhibited significantly lower GUS activity in all organs, with some localized expression being seen in the cotyledons; more so in the veins than the other tissues (Fig. 5 C1-C4). Deletion of the 5’UTR + ORF (*AtSAMDC*3-C and D) had little effect on *GUS* expression in the roots. For *AtSAMDC*4, the effect of ORF was just the opposite of that in *AtSAMDC*3; more GUS activity was seen in its presence than its absence (Fig. 5 D1-D4). A pattern similar to that of *AtSAMDC*3 was observed for *AtSAMDC*5 constructs where presence or absence of 5’UTR did not affect GUS activity in the seedlings (Fig. 5 E1-E3).

### Activity of different promoter constructs in mature vegetative organs

**Fig. 6:**
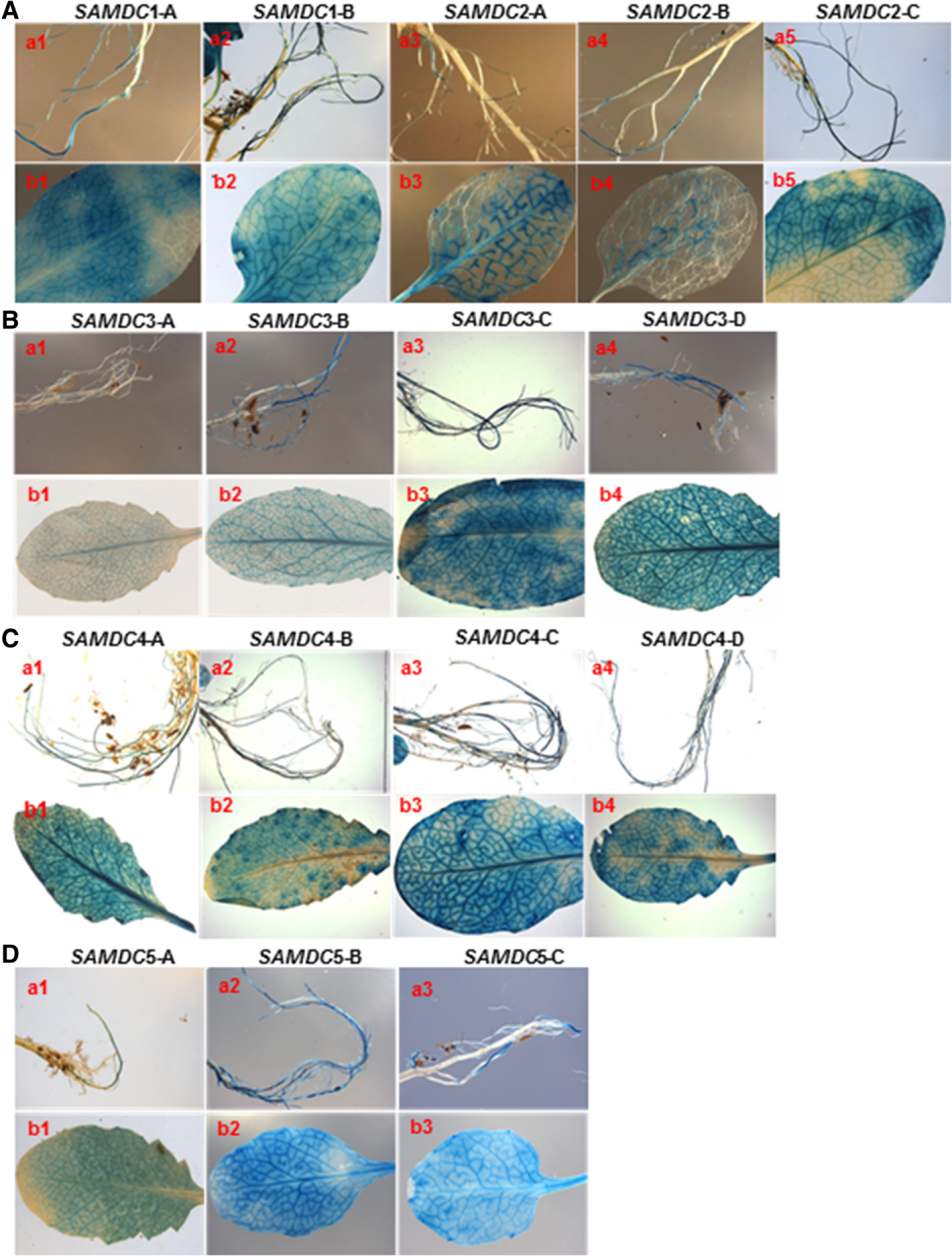
Activity of GUS in roots and rosette leaves of 5-week old transgenic plants. **a** *AtSAMDC*1 and *AtSAMDC*2; **b** *AtSAMDC*3; **c** *AtSAMDC*4;  **d** *AtSAMDC*5. Roots (**a**: a_1–5,_ **b**: a_1–4,_ **c**: a_1–4,_ **d**: a_1–3_); and rosette leaves (**a**: b_1–5,_ **b**: b_1–4,_ **c**: b_1–4,_ **d**: b_1–3_)

The activity of *GUS* under different promoters varied widely in the roots of mature plants in terms of intensity of the blue color as well as distribution in the main root vs. the secondary roots (Fig. 6). The effects resulting from the presence or absence of the 5’UTR and the ORF were also variable. The intensity of blue color in the roots was often quite low. In all constructs, GUS appeared to be expressed quite well in the rosette junctions, again the exception being *AtSAMDC*2-A, where the ORF was present (Additional file 1: Fig. S5). All promoters showed high activity in the rosette leaves, although there were differences in GUS activity with respect to its distribution in the vascular tissues vs. other tissues in the lamina; mostly a reduction in GUS activity was associated with the presence of the 5’UTR and the ORF. Often the shorter promoter sequences showed more activity than the longer ones. In contrast to the rosette leaves, the cauline leaves often showed lower GUS activity, and its distribution was more diffused than that in rosettes leaves (Additional file 1: Fig. S6). High activity of GUS was present for *AtSAMDC*1 and *AtSAMDC*2 constructs in the leaf and stem trichomes, and hydathodes [Additional file 1: Fig. S5 (b1-b3)]. For all constructs, significant GUS activity was observed in the secondary roots and root junctions, elongation zone, root hairs, root tips and lower part of the primary roots. No consistent pattern of activity in relation to different promoter constructs could be established.

### Activity of different promoter constructs in reproductive parts

**Fig. 7:**
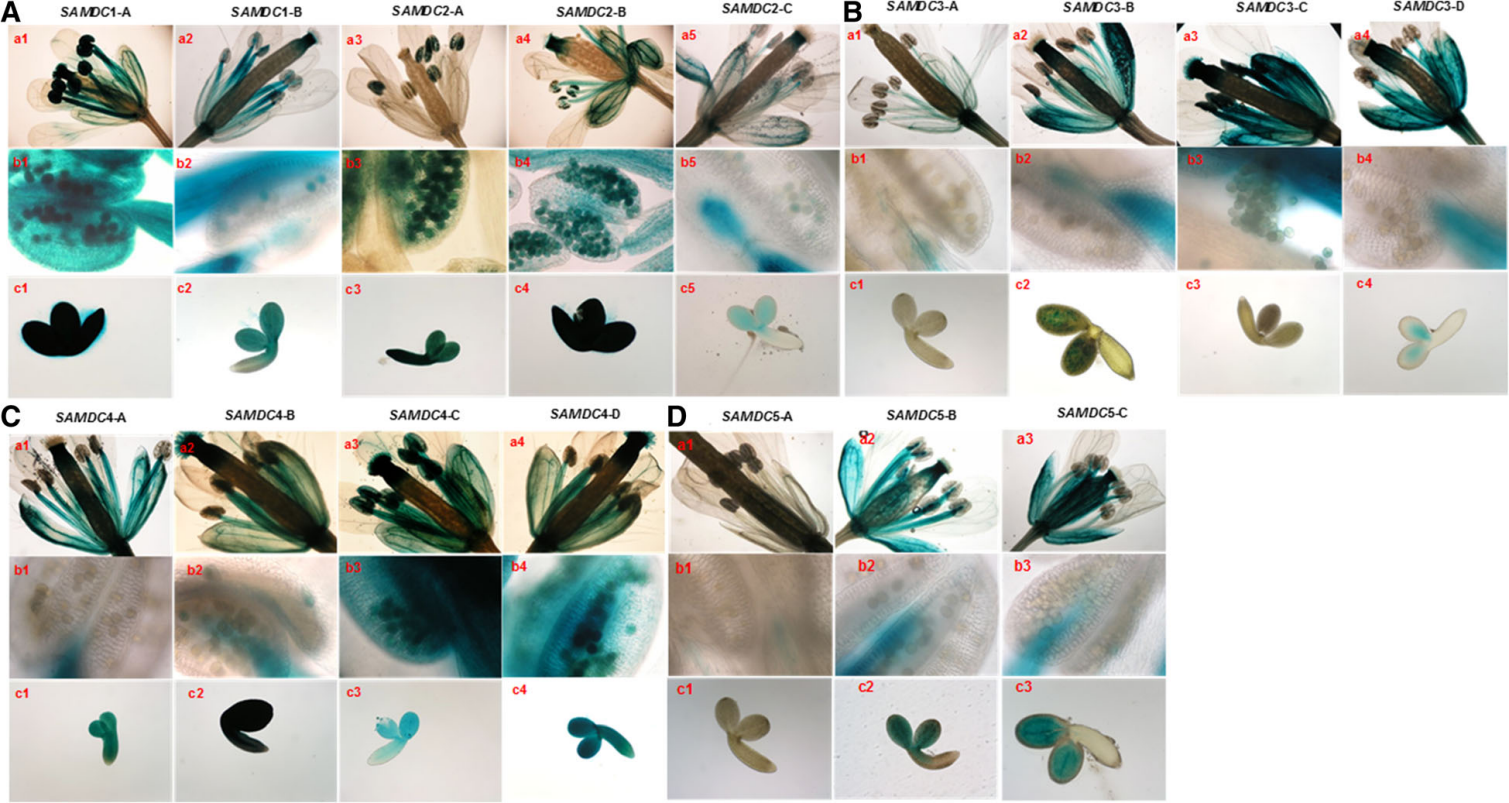
Activity of GUS in reproductive parts of mature transgenic plants. **a** *AtSAMDC*1 and *AtSAMDC*2; **b** *AtSAMDC*3; **c** *AtSAMDC*4; **d** *AtSAMDC*5. Flower (**a**: a_1–5,_ **b**: a_1–4,_ **c**: a_1–4,_ **d**: a_1–3_); close up view of anther sac and pollens (**a**: b_1–5,_ **b**: b_1–4,_ **c**: b_1–4,_ **d**: b_1–3_); and embryo (**a**: c_1–5,_ **b**: c_1–4,_ **c**: c_1–4,_ **d**: c_1–3_)

High activity of GUS was observed in the inflorescence stalks and unopened flower buds of *AtSAMDC*1 plants. The intensity of blue color was highest in the stamens particularly in the pollen grains (Fig. 7A), besides high activity in the stigma, receptacle, sepals and petals (mainly in the veins). In contrast to *AtSAMDC*1, *AtSAMDC*2 showed relatively low activity of GUS in most flower parts, except for the sepals, un-opened flower buds, and inflorescence stalks that showed high GUS activity. In *AtSAMDC*2-A flowers, high and localized activity of GUS was observed in the pollen grains with very low expression in the sepal veins (Fig. 7A). On the other hand, *AtSAMDC*2-B construct showed highest GUS activity in the stamens (again more so in the anthers and pollen grains), but less in the sepals and the stigma. Deletion of a major part of the promoter reduced overall GUS activity in the flowers of *AtSAMDC*2-C construct. The activity of GUS was quite low in the *AtSAMDC*3-A flowers, and it was mainly localized in the sepal veins, petals, filaments and the receptacle (Fig. 7B). In the flowers of *AtSAMDC*3-B (plus 5’UTR but no ORF) plants, GUS activity increased (with further increase in *AtSAMDC*3-C and *AtSAMDC*3-D constructs) significantly in the same organs, with no expression in the anther sacs or the ovary wall (Fig. 7B). No activity of GUS was detected in the developing embryos of *AtSAMDC*3-A and *AtSAMDC*3-D plants, whereas light blue color was observed in the cotyledons of developing embryos. High GUS activity was also seen in the upper part of the ovary including the base of the stigma, except in *AtSAMDC3*-A plants. Reproductive tissues and organs showed high activity of GUS in the inflorescence stalks and unopened flower buds of *AtSAMDC*4::*GUS* plants. In *AtSAMDC*4-A mature flowers, GUS activity was highest in the stamens; particularly in the filaments (with little expression in the anther sac) and in the sepal veins (Fig. 7C). Stigma and receptacle also showed higher GUS activity but ovary wall had little activity. In *AtSAMDC*4-B flowers, high activity of GUS was observed in all parts with some activity being seen in the anther sac cells but pollen did not stain blue. Removal of 5’UTR increased overall GUS activity in *AtSAMDC*4-B and *AtSAMDC*4-C flowers; GUS was also detected in the pollen grains/microspores (Fig. 7C). High activity of GUS was observed for all *AtSAMDC*4 constructs throughout the developing embryos’ cotyledons. Unlike the *AtSAMDC*4::*GUS* plants, the flowers of *AtSAMDC*5-A::*GUS* plants showed only a little or no GUS activity in any part (Fig. 7D). The intensity of blue color increased significantly in *AtSAMDC*5-B and *AtSAMDC*5-C without any activity being seen in the petals or pollen grains. No activity of GUS was detected in the embryos of *AtSAMDC*5-A and the siliques, whereas low levels of GUS activity were observed in the developing embryos of *AtSAMDC*5-B and *AtSAMDC*5-C.

In general, for developing siliques, high activity of GUS was common in the silique tips, pedicel and the valve junctions for all *AtSAMDC*s; the intensity of blue color and its distribution varied among different constructs of the same gene, and among the different genes (Additional file 1: Fig. S7).

### *AtSAMDC* gene expression, SAMDC activity, and polyamine contents

**Fig. 8:**
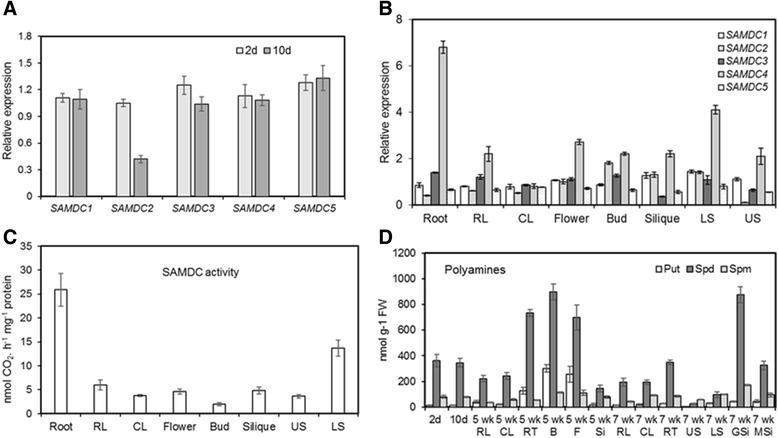
Gene expression, enzyme assay, and polyamine contents in wild type *Arabidopsis thaliana*. **a** relative expression of the five *AtSAMDC* genes (white bars = 2-day and gray bars = 10-day old seedlings; **b** *AtSAMDC* gene expressions in 5-week old mature plant parts; **c** SAMDC enzyme activity in 5-week old mature plant parts; and (**d**) polyamine contents in seedlings and mature plant parts (abbreviations used: RL = rosette leaf, CL = cauline leaf, 2 d = 2 day whole seedlings, 10 d = 10 day whole seedlings, wk. = week, RT = root, F = flower, B = bud, LS = lower stem, US = upper stem, GSi = green silique, MSi = mature silique). Data are mean ± SE of 3–4 biological replicates

In order to establish, (1) if the promoter::*GUS* fusion results revealed a similarity of expression with native transcript levels of the respective gene at various stages of seedling development or in different parts of the mature plant, for various *AtSAMDC* genes, and (2) to identify the promoter construct(s) that would mimic the actual expression of a particular *AtSAMDC* gene, QRT-PCR analysis was performed for each gene in selected organs of the WT plants. Relatively high and comparable levels of transcripts were observed in seedlings at initial stages (e.g. 2 d after germination) for all five *AtSAMDC* genes (Fig. 8A). However, by 10 d after germination, the seedlings began to show differences, particularly for the *AtSAMDC*2 gene, whose relative expression was about half that of the other four genes (Fig. 8A). In 5-week old plants, the expression of *AtSAMDC*4 was the highest; its transcripts in the root and the lower part of the stem being 2- to 7-fold higher than other *AtSAMDC* genes. On the other hand, transcripts of all *AtSAMDC* genes were present at all times, and in all organs of mature plants (Fig. 8B).

Among different organs studied, roots showed the highest SAMDC activity, which was several-fold greater than the other organs (Fig. 8C). Other organs showed little variation of SAMDC activity among each other with rosette leaves and lower stem having slightly higher SAMDC activity than other organs.

Polyamine analyses of tissues collected from WT plants at different developmental stages showed Spd as the predominant PA in all tissues (146–362 nmol/g Fresh weight - FW) and at all times with relatively higher contents (699–896 nmol/g FW) in the flower buds, flowers and green siliques (Fig. 8D). Of the three PAs, Put was the lowest in most of the organs, ranging between 0 to 46 nmol/g FW except for reproductive organs, e.g. flowers, flower buds (250–300 nmol/g FW) and roots (~130 nmol/g FW) of 5-wk. old plants that maintained relatively high contents of Put. Spermine contents were slightly higher than Put in most organs (36–96 nmol/g FW), except for flowers and buds that had higher amounts of Put than Spm.

## Discussion

### The polyamine biosynthetic genes in plants are highly conserved

Gene families in plants or other eukaryotic organisms often comprise of gene members sharing common nucleotide sequences, arising from various gene duplication events, and showing varying degrees of functional redundancy [57, 58]. The number of members within a gene family in higher plants can vary anywhere between 2 to >40; the size variation among gene families is often attributed to speciation and adaptation to the changing environment. Extensive studies with several gene families in plants suggest that genetic divergence and functional diversity among gene family members could be a consequence of variation in the promoter region resulting from spatial distribution and frequencies of transcription factor binding sites within the promoter regions. Besides promoter evolution, functional diversity and divergence of gene family members could also arise from varying degrees of base substitutions in the 5’UTR, the protein coding region, or the 3’UTR regions. [58, 59].

A closer look at the key PA biosynthetic genes (*ADC*, *ODC*, *SPDS* and *SPMS/tSPMS*) in plants shows that (1) all of them have remained highly conserved among different species (except that *ODC* is absent in *A. thaliana*), (2) they typically have only two copies each, except one copy each of *SPMS* and *tSPMS* (3) their promoters have several regulatory motifs common to each other (MYB4 binding site motif, GAREAT, W-box, ARF, etc.), and (4) they are expressed mostly in a redundant manner in different tissues and organs [60–65]. While *AtSAMDC* gene family appears to follow many of the same attributes as other genes whose products are involved in the PA biosynthetic pathway, it differs from them in several ways including the facts that there are as many as five members of the *AtSAMDC* gene family with a rather complex mechanism of regulation for its transcription as well as its translation. In this regard, it seems to follow the situation of *AtPAO*s, which also have five members [66], with one big difference that while the SAMDC function is highly specific with respect to its substrate, the nomenclature and the function(s) of PAOs are not as specific as that of SAMDC [67, 68]. The existence of multiple gene copies of a critically important enzyme involved in a single metabolic reaction without the necessity for hetero-di/polymer formation, raises important questions about: (a) the importance of redundancy and complementation of gene expression in different tissues and organs, and (b) the co-evolution vs. independent evolution of the different components of the gene; i.e. the promoter, the coding sequences, and/or the 5′ and 3’ UTRs - in the present case including the uORFs within the 5’UTRs. The SAMDC is among one such enzyme which remains indispensable for all living organisms.

Here we suggest a few answers to the above questions using a combination of bioinformatics analyses, and extensive experimental analyses of the five known members of *AtSAMDC* gene family in *A. thaliana*. The answers seem to be rather complex in that while the coding sequences of the enzyme as well as the promoter regions and uORFs (where present) have stayed highly conserved within the family, the 5’UTRs and 3’UTRs themselves have followed quite different evolutionary paths. What is surprising is that the evolution has not led to any major compartmentalization of expression in a cell/tissue/organ or a developmental stage-specific manner since most of the paralogs are expressed in most of the tissues/organs concurrently. This, however, does not rule out the possibility of regulation of their expression in response to various environmental stimuli; e.g. abiotic or biotic stress.

Comparisons of CDSs among different members of the *AtSAMDC* gene family members showed 46–78% sequence identities whereas, the sequence identities among promoters ranged between 39 and 50%. Is it that the evolution of a promoter results in differential expression or the need for differential expression leads to evolutionary path of the promoter? The diversity of the promoter regions of the five genes is apparent from their size as well as the size of their 5’UTRs, which have a translation regulatory function [1, 8, 9]. In terms of the evolutionary nearness among different *AtSAMDC* gene coding sequences, *AtSAMDC*1 and *AtSAMDC*2 apparently evolved at the same time and *AtSAMDC*3 and *AtSAMDC*4 were the closest, while *AtSAMDC*5 might have diverged from either of these two groups. The upstream promoter elements followed a parallel pattern of divergence as the coding sequences among different *AtSAMDC* genes. Furthermore, genetic divergence of 5’UTRs and 3’UTRs was independent of the upstream elements and the coding sequences. This might implicate a major role of 5’UTR evolution resulting in genetic divergence and functional diversity among the different *AtSAMDC* genes.

Tajima test (43) revealed that promoters followed positive selection while UTRs exhibited some rare variants. We did not conduct the Tajima test with SAMDC coding sequences because the test assumptions do not hold true with coding sequences due to the extent of polymorphism associated with first, second and third codons as well as codon usage biases [69]. Tajima test has been conducted on non-coding sequences in other studies to test the type of selection [70, 71].

### Benefits of redundancy vs. division of labor

The expression data of all *AtSAMDC* genes during early development show that even though the germinating seedlings start with expression of all *AtSAMDC*s, there certainly is a differential role of the various *AtSAMDC* gene family members in different tissues and organs as the plant develops and matures. Furthermore, the published data on *AtSAMDC* expression in response to abiotic stress treatments confirm that not only might they play differential roles during development; they also respond differently to environmental stress factors [65]. These conclusions are consistent with the evolutionary changes in different members of large gene families where they may have arisen from gene duplication and rearrangements [72, 73]. The greater differences in sequences of the different promoters vs. the coding sequences further indicate that the promoter sequences perhaps evolved faster than the CDSs, due to the highly conserved function of the enzyme. On the other hand, it can also be argued that the promoter regions of all *AtSAMDC* genes must share certain common regulatory elements that control the basic function of regulating gene expression per se (the core promoter functions), and others that control its differential expression in response to common environmental signals (e.g. different forms of abiotic stress) in a tissue/organ specific manner in different parts of the mature plant. This is evident from the presence of several MYB transcription factor binding sites e.g. MYB1AT, MYB4 that are associated with dehydration, drought, salt, cold, and wounding stresses that were common among all the *AtSAMDC* genes. Besides common motifs, certain unique motifs e.g. ARF (auxin responsive), BoxII (transcriptional activator), CCA1 (phytochrome regulation), MYB1LEPR (defense related genes), were observed only in *AtSAMDC*3, *AtSAMDC*4 and *AtSAMDC*5 (Table 4, Additional file 1: Tables S4-S8) genes suggesting their probable interaction with hormones and other environmental stimuli during plant growth and development. This is borne out from the distribution of various *cis*-regulatory elements in the promoters. Careful observation of the relative distribution of transcription factor binding sites (within 700 bp upstream of transcription start site) among the different *AtSAMDC* genes reveal a distinct pattern where a higher frequency of transcription factor binding sites were observed within 600 bp upstream of the transcription start site in *AtSAMDC*1, *AtSAMDC*2, and *AtSAMDC*3 whereas, it was between 400 and 600 bp upstream of the transcription start site in *AtSAMDC*4 and *AtSAMDC*5*.*

The different translational and transcriptional fusions with the *GUS* gene used in the present study provide a deeper insight into the regulation of *AtSAMDC* genes at different levels of gene expression and at different stages of development. Higher activity of GUS for the translational fusion of *AtSAMDC*4 and *AtSAMDC*1 were observed during the first 48 h of seed germination in all parts of the seedlings. On the other hand, little or no GUS activity for *AtSAMDC*2::*GUS* and *AtSAMDC*3::*GUS* was observed, and *AtSAMDC*5::*GUS* showed only limited activity in the cotyledons. This suggests a prominent role of the native *At*SAMDC1 isomer of the enzyme at early stages of seedling development; and a differential regulation of its activity at the translational level by the uORF. Increased expression on deletion of the 5’UTRs in all three *AtSAMDC*s, further confirms the involvement of 5’UTR in translational regulation of the *AtSAMDC* genes as reported earlier [8, 74, 75]; even though the size of the 5’UTR varies considerably among them.

Deletion of a major 5′ portion (up to 80% in some cases) of the putative promoter in all *AtSAMDC*s (leaving only 384 bp promoter sequence in *AtSAMDC*1, *AtSAMDC*2, *AtSAMDC*3, and *AtSAMDC*5, and 283 bp in *AtSAMDC*4) did not negatively impact GUS activity; instead, there was enhanced GUS activity both in vegetative and reproductive organs with the minimal promoter sequence. This would suggest that the core promoters for all *AtSAMDC*s are much smaller to drive sufficient spatio-temporal pattern of gene expression; and the remaining 5′ parts of the promoters with several common motifs are associated either with developmental and environmental responses or with fine-tuning of gene expression in different cell types within a tissue/organ. As an example, the presence of GAREAT (GA Response Element) motifs in the promoter of *AtSAMDC*3 and *AtSAMDC*5, and their higher expression in the transcriptional fusions, is consistent with the role of GA in seed germination. The expression data during early development for *AtSAMDC*3 and *AtSAMDC*4 are consistent with the reported patterns using microarrays (www.genevestigator.com). Lack of expression data on *AtSAMDC*5 in the published literature leads to a suspicion that this gene might not be transcribed to produce a functional protein. However, the data presented here clearly demonstrate that the *AtSAMDC*5 promoter is transcriptionally active, and it’s minimal 5’UTR (12 bp) may still (negatively) regulate its translation, since the removal of 5’UTR further increased the activity of GUS.

In mature organs, including flowers, whereas *AtSAMDC*1 promoter showed constitutively high GUS activity, the *AtSAMDC*2 promoter exhibited a localized pattern of *GUS* expression, limited largely to leaf veins and hydathodes. In contrast to *AtSAMDC*1 and *AtSAMDC2*, no activity of GUS for *AtSAMDC*3, *AtSAMDC*4 (except in the constructs with promoter in absence of 5’UTR and ORF) and *AtSAMDC*5 was detected in the pollen, which clearly points to the differential expression of different members of this gene family in flower parts. The GUS activity profiles for both *AtSAMDC*1 and *AtSAMDC*2 in flowers are consistent with the available microarray data in that highest signal values were obtained for pollen followed by sepals, petals, stigma and ovary, respectively, for both; and, overall expression of *AtSAMDC*1::*GUS* was much greater than *AtSAMDC*2::*GUS*. High activity of GUS in developing embryos of *AtSAMDC*1, *AtSAMDC*2 and *AtSAMDC*4 also reinforces earlier findings [14], where homozygous *samdc1*/*bud2* (*AtSAMDC*4) double mutant showed embryo lethality.

Again, at the organ/tissue level, high activity of GUS in the vascular tissues of leaves, roots, and rosette junctions in translational fusion of *AtSAMDC*4, and its near absence for *AtSAMDC*3 and *AtSAMDC*5 fusions agrees with earlier reports on the expression of at least three *AtSAMDC*s [1, 11, 14], and the micro-array data (except for *AtSAMDC*5) for several organs in *A. thaliana* (www.genevestigator.com). High level of *AtSAMDC* expression in the vascular tissues perhaps is related to intense cell division activity associated with this tissue. It is quite possible that large amounts of PAs are actually produced in the vascular tissue and transported to many cells/tissues that do not show *SAMDC* expression. A role of PAs in plant vasculature development is implicated in several plant species [76, 77]. A possible explanation for PA role in vasculature differentiation and lignification is through the production of H_2_O_2_ via PA oxidation by DAOs and PAOs [68, 76–79]. The involvement of *SAMDC* in the development of proper vasculature was demonstrated in *bud2* (a mutant for *Atsamdc4*), which had limited dcSAM availability, and showed increased vascular bundle size [14]. Altered hypocotyl elongation and lateral bud growth in *bud2* was later explained as an effect of altered auxin-mediated response in this mutant [80]. Bioinformatics analysis of the promoters of all *AtSAMDC* genes further supports interaction of auxin with *AtSAMDC*4 [80]; the *AtSAMDC*4 along with *AtSAMDC*5 promoters show auxin responsive elements in their promoter regions.

Biosynthesis of higher PAs and another important phytohormone ethylene, is mediated by SAM (a universal methyl group donor in the cells) in plants that serves as the common precursor of both PAs and ethylene [81]. Besides the role of ethylene in fruit ripening and senescence, it is also involved in active cell division (in coordination with auxin) and shape determination in plants [82]. Ethylene is known to be actively involved in root and flower development, and stress responses in plants; features that are common to those of PAs. Presence of auxin responsive elements in some *AtSAMDC* promoters (*SAMDC*4 and *SAMDC*5; Table 4) might also indicate a coordinated action of PAs, auxin, and ethylene during Arabidopsis growth and development. As both ethylene (via ACC) and dcSAM share the common precursor SAM, high co-expression of *SAMDC* genes and ethylene biosynthetic genes at specific developmental stages (such as root development, floral initiation) and in certain tissues (e.g. vascular tissues) would require increased biosynthesis of SAM (from methionine – Met by SAM synthetase to feed these two pathways. This would also require co-operation between PA biosynthesis and ethylene biosynthesis rather than a competition (such as antagonistic roles of PAs and ethylene during senescence) between these two pathways that might be developmentally regulated or spatially separated [83, 84]. Ethylene is also reported to induce H_2_O_2_ production (and its role in physiological responses) by the increased expression and activities of PAOs in plants. In context to the recycling of dcSAM, the bi-product, 5′-deoxy-5′-methylthioadenosine (MTA; derived from dcSAM) produced during the biosynthesis of Spd and Spm, is recycled back to Met (Met salvage) by the genes involved in the Yang cycle [85–88]. Significantly high co-expression of the *SAMDC* genes and Yang cycle genes (along with higher Yang cycle intermediates) in the vascular tissues of Arabidopsis show that there is a high demand of Spd (evident from significant accumulation of SPDS in the phloem); [89] and Spm in these tissues and rapid recycling of MTA into Met might be required to maintain cellular homeostasis of SAM.

### Correlation among gene expression, enzyme activity and polyamines

A lack of strong correlation between gene expression and the activity of the corresponding enzyme is often observed in biological studies, as gene expression (i.e. transcription) and the production of the enzyme protein (translation) are regulated differently before the production of an active enzyme. This involves complex transcriptional and post-transcriptional controls at the mRNA as well as translational levels [8, 9, 74]. Moreover, in many cases, the protein itself may have to be further modified to become a functional enzyme. Likewise, enzymatic controls over the cellular contents of the resulting metabolites are also complex and mostly indirect, involving the availability of substrates, the end-product catabolism, the availability of appropriate co-factors, and feed-back inhibition to name a few [28, 81, 82, 90, 91]. At individual reaction level also, metabolites often show non-linear correlation with the corresponding enzyme abundance. Thus, metabolic network connectivity has been attributed as the primary control over the metabolite levels in living cells [90]. Our data show (Fig. 8) that the native transcript abundance of individual *AtSAMDC* genes strongly correlates with the GUS activity of the corresponding *AtSAMDC* transcriptional fusion constructs at the organ level, but not with the individual SAMDC proteins at the cellular/tissue levels (since quantification of the individual SAMDC proteins and their enzymatic activity are not possible to quantify at present). The results presented here illustrate the complexity of interactions between gene expression, translation, and metabolite production in plants during development.

## Conclusions

Differential role of positive/purifying selection was observed in genetic divergence of the *AtSAMDC* gene family. While promoter sequences overall showed a strong positive selection, UTRs or protein coding sequences did not always follow the same pattern. It was seen that all tissues express one or more *AtSAMDC* genes with significant redundancy; at the same time, there is specificity of gene expression, particularly in mature organs. The *AtSAMDC*5, which is believed to be inactive, is transcriptionally active, and is a unique member of the family in that its expression is not regulated by 5’UTR. Overall, there is differential regulation of the *AtSAMDC* gene family members at the level of transcription and translation to produce higher PAs in a spatio-temporal manner. This study provides valuable information about At*SAMDC* promoters, which could be useful in the manipulation of crop plants for nutritive purposes, stress tolerance or bioenergy needs.

## Additional file

Additional file 1: Tables S1-S8; Figs. S1-S7. (DOC 3393 kb)

## Abbreviations

ADC: arginine decarboxylase
Arg: arginine
CDS: coding sequence
DAO: diamine oxidase
FW: fresh weight
GM: germination medium
HPLC: high performance liquid chromatography
IAA: indole-3-acetic acid
Met: methionine
ODC: ornithine decarboxylase
Orn: ornithine
PA: polyamines
PAO: polyamine oxidase
Put: putrescine
*SAMDC*: S-adenosylmethionine decarboxylase
SE: standard error
Spd: spermidine
SPDS: spermidine synthase
Spm: spermine
SPMS: spermine synthase
uORF: upstream open reading frame
UTR: untranslated region
WT: wild type
