# Supplementary material for: Polyamines in the life of Arabidopsis: profiling the expression of S-adenosylmethionine decarboxylase (SAMDC) gene family during its life cycle
Source: BMC Plant Biol. 2017 Dec 28;17:264. doi: 10.1186/s12870-017-1208-y (PMC5745906; doi:10.1186/s12870-017-1208-y)
Supplement: Additional file 1: — Tables S1-S8; Figs. S1-S7. (DOC 3393 kb) [file 12870_2017_1208_MOESM1_ESM.doc]

**Additional files**

**Additional file 1: Table S1.** Comparison of the % sequence identities among different members of the Arabidopsis *SAMDC* gene family. **Table S2.** Primers used for cloning, sequencing and (Q)RT-PCR. (Q)RT-PCR primers of *AtSAMDC*1-4 were adapted from [17]. **Table S3.** Genomic organization of *SAMDC* genes from different plant species those are phylogenetically related to *Arabidopsis thaliana* *SAMDC* [11, 5]. Abbreviations used: *At= Arabidopsis thaliana*; *Os= Oryza sativa*; *St= Solanum tuberosum*; *Dc= Daucus carota*; *Zm= Zea mays*; *Bj= Brassica juncea*; *Dec= Dendrobium crumenatum*; *Ps= Pisum sativum*; *Vf= Vicia faba*; *So= Spinacia oleracia; Dic= Dianthus caryophyllus*; *Gm= Glycine max*; *Hv= Hordeum vulgare*. **Table S4.** Putative *cis* elements in the *AtSAMDC*1 promoter sequence [analyzed using Athena promoter analysis software, 46] showing consensus sequences, binding sites, and physiological responses of the identified elements. **Table S5.** Putative *cis* elements in the *AtSAMDC*2 promoter sequence [analyzed using Athena promoter analysis software, 46] showing consensus sequences, binding sites, and physiological responses of the identified elements. **Table S6.** Putative *cis* elements in the *AtSAMDC*3 promoter sequence [analyzed using Athena promoter analysis software, 46] showing consensus sequences, binding sites, and physiological responses of the identified elements. **Table S7.** Putative *cis* elements in the *AtSAMDC*4 promoter sequence [analyzed using Athena promoter analysis software, 46] showing consensus sequences, binding sites, and physiological responses of the identified elements. **Table S8.** Putative *cis* elements in the *AtSAMDC*5 promoter sequence [analyzed using Athena promoter analysis software, 46] showing consensus sequences, binding sites, and physiological responses of the identified elements. **Figure S1.** Phylogenetic analyses of the CDSs of Arabidopsis *SAMDC* orthologs. **Figure S2.** Phylogenetic analyses of the 5’UTRs of Arabidopsis *SAMDC* orthologs. **Figure S3.** Comparison of the upstream (700 bp) promoter regions between different *SAMDC* genes of Arabidopsis using GATA promoter comparison software [41]. Similar regions are connected by solid black lines and dotted red lines connect matched regions in reversed orientation. **Figure S4.** Transient expression of different constructs of *AtSAMDC* genes in poplar cells. The data represented here are from 3-6 independent shootings and expressed as % blue cells as compared to the control plasmid pCAM-*35S*::*GUS*. **Figure S5.** Activity of GUS in rosette junctions of 5-week old *AtSAMDC* transgenic plants. Stem and leaf trichomes of *AtSAMDC*1-A plants (b1), leaf hydathodes and trichomes of *AtSAMDC2*-A (b2, b3) plants. Similar GUS activity was observed in the *SAMDC2*-B plants. **Figure S6.** Activity of GUS in the siliques of 5-week old *AtSAMDC* transgenic plants.

Additional Data available from the Dryad Digital Repository: [https://doi.org/10.5061/dryad.457fc](https://urldefense.proofpoint.com/v2/url?u=https-3A__doi.org_10.5061_dryad.457fc&d=DwMFaQ&c=c6MrceVCY5m5A_KAUkrdoA&r=sgWyvmMlfRAcpYN-oC433_EV_BBPNuHpsgXvnpWkOeo&m=BwhzGmOmCiLoo5n43-mbmip3wUSHA0ZnsBeXJL49hEg&s=k-V0FK2uIXFns2BH7Kuw3FNND9ou8We3TNVe-HI89no&e=)

Title of Data: Data from: Polyamines in the life of Arabidopsis: profiling the expression of S-adenosylmethionine decarboxylase (SAMDC) gene family during its life cycle

**Table S1** Comparison of the % sequence identities among different members of the Arabidopsis *SAMDC* gene family.

| **Genes compared** | **Promoter** | **5’UTR** | **Coding sequence** | **3’UTR** | **Genomic**  (5’UTR+ORF+3’UTR) |
| --- | --- | --- | --- | --- | --- |
| *AtSAMDC*1*/ AtSAMDC*2 | 46.2 | 46.5 | 78.1 | 44.1 | 61.6 |
| *AtSAMDC*3*/*  *AtSAMDC*4 | 44.0 | 7.2 | 57.5 | 25.4 | 33.7 |
| *AtSAMDC*1*/ AtSAMDC*3 | 49.0 | 47.4 | 63.1 | 46.5 | 54.3 |
| *AtSAMDC*2*/ AtSAMDC*3 | 42.4 | 48.2 | 63.9 | 49.2 | 57.9 |
| *AtSAMDC*1*/ AtSAMDC*4 | 42.7 | 6.1 | 53.4 | 30.4 | 31.5 |
| *AtSAMDC*2*/*  *AtSAMDC*4 | 44.9 | 7.9 | 52.9 | 25.5 | 35.3 |
| *AtSAMDC*1*/ AtSAMDC*5 | 48.2 | 0.8 | 50.8 | 18.6 | 27.9 |
| *AtSAMDC*2*/ AtSAMDC*5 | 39.1 | 1.0 | 50.2 | 22.0 | 32.6 |
| *AtSAMDC*3*/ AtSAMDC*5 | 50.3 | 1.1 | 45.6 | 24.8 | 33.1 |
| *AtSAMDC*4*/ AtSAMDC*5 | 42.1 | 7.4 | 48.3 | 10.3 | 43.1 |

**Table S2** Primers used for cloning, sequencing and (Q)RT-PCR. (Q)RT-PCR primers of *AtSAMDC*1-4 were adapted from [17].

| **Constructs** | **Forward (5’-3’)** | **Reverse (5’-3’)** | **Td (ºC)** |
| --- | --- | --- | --- |
| *AtSAMDC*1-A | GGGCCCTTTTCATCATATTGTCAAGTTGGGT | GTCGACCTGCTTGGCTCAAAGAAAGTCACCT | 59.0 |
| *AtSAMDC*1-B | TTTATATTTTGTTGTTCCGCA | GGTAATGATGAAGCAATGAG | 54.0 |
| *AtSAMDC*2-A | CGAAGGTCGTCTAGTAAA | GGATCCAGGCTCAAAGAAAGTAACT | 46.0 |
| *AtSAMDC*2-B | CGAAGGTCGTCTAGTAAA | AAGCTTCTCTGCGTTGTTGTTGT | 46.0 |
| *AtSAMDC*2-C | TCTTTAAATACATTTTGACAAAA | GTAGAGAAGAAGGTGGAGGG | 54.0 |
| *AtSAMDC*3-A | CTTGCTTGCCATAACTGCGA | GAACCCAAGAGTAGATTTTGGTGA | 62.0 |
| *AtSAMDC*3-B | CTTGCTTGCCATAACTGCGA | TTTTCAAAAGGCGAAGAGACT | 59.0 |
| *AtSAMDC*3-C | TCTACATTAATAATAACCTCTAATG | TGAAGAACAGAGCAATAAAAAG | 54.6 |
| *AtSAMDC*3-D | GTTCAATCTAATAAAGGGTAAATC | TGAAGAACAGAGCAATAAAAAG | 54.6 |
| *AtSAMDC*4-A | ATGATTGGTAGCTTTTGAAGCGA | TTTCCGACGAGGCGTGAAGG | 63.6 |
| *AtSAMDC*4-B | ATGATTGGTAGCTTTTGAAGCGA | TGCAAAGATCAGAAGAAGATGTTATG | 63.0 |
| *AtSAMDC*4-C | ATGATTGGTAGCTTTTGAAGCGA | TGCAAAGATCAGAAGAAGATGTT | 59.0 |
| *AtSAMDC*4-D | CTCTCACATACACAGATACATACAC | TGCAAAGATCAGAAGAAGATGTT | 54.0 |
| *AtSAMDC*5-A | GAGCACTTTTGGAGCAGTAAT | GACAGCCAAGAATGTAAAAAA | 55.5 |
| *AtSAMDC*5-B | GAGCACTTTTGGAGCAGTAAT | TGGAATTGAATTAGAGAGAAA | 52.0 |
| *AtSAMDC*5-C | AATTAACCGGTCGGGTCG | AGAGAGAAACGAGAGAGATAGATT | 58.5 |
| pCR8.0 TOPO (sequencing) | GTTGCAACAAATTGATGAGCAATGC | GTTGCAACAAATTGATGAGCAATTA | 50.0 |
| *AtSAMDC*1 (QRT-PCR) | TCGAGCCCAAGCAATTCTCT | CAAATGTCCTCTCTCTGCACCC | 60.0 |
| *AtSAMDC*2 (QRT-PCR) | ACCATTCCCTCACCGCAACTT | GGTTTCATCGTCATTGCCCAT | 60.0 |
| *AtSAMDC*3 (QRT-PCR) | ACCCCGGAAGATGGTTTTAGC | GCCAATACCTCGGTTCCAAGA | 60.0 |
| *AtSAMDC*4 (QRT-PCR) | ACAACCACGAGGTGACTAAGCG | GGCGTGAAGGACTGATAAACGA | 60.0 |
| *AtSAMDC*5 (QRT-PCR) | GGTACGACTCAACAATGGTTTATC | TGCACATCTCAATCGTTGGTAG | 60.0 |
| *AtTIP*41 (QRT-PCR) | CGAGGTTTACGCATCCATGA | TCGACAGCGAGAGAAGTGAGAA | 60.0 |

**Table S3** Genomic organization of *SAMDC* genes from different plant species those are phylogenetically related to *Arabidopsis thaliana* *SAMDC* [5, 11]. Abbreviations used: *At= Arabidopsis thaliana*; *Os= Oryza sativa*; *St= Solanum tuberosum*; *Dc= Daucus carota*; *Zm= Zea mays*; *Bj= Brassica juncea*; *Dec= Dendrobium crumenatum*; *Ps = Pisum sativum*; *Vf= Vicia faba*; *So= Spinacia oleracia; Dic = Dianthus caryophyllus*; *Gm = Glycine max*; *Hv= Hordeum vulgare*.

| **Genus** | **5’UTR (bp)** | **Tiny uORF (bp)** | **Small uORF (bp)** | **ORF (bp)** | **3’UTR (bp)** |
| --- | --- | --- | --- | --- | --- |
| *AtSAMDC*1 | 1106 | 691-702 (12) | 702-865 (156) | 1101 | 238 |
| *AtSAMDC*2 | 837 | 553-564 (12) | 564-719 (156) | 1089 | 193 |
| *AtSAMDC*3 | 876 | none | 468-635 (168) | 1050 | 180 |
| *AtSAMDC*4 | 108 | none | none | 1044 | 447 |
| *AtSAMDC*5 | 12 | none | none | 1164 | 52 |
| *OsSAMDC*1 | 539 | 197-205 (9) | 205-360 (156) | 1197 | 300 |
| *OsSAMDC2* | 536 | 212-220 (9) | 220-378 (156) | 1188 | 247 |
| *StSAMDC* | 1570 | 1105-113 (9) | 1113-1248, 1354-1374 (156) | 1083 | 251 |
| *DcSAMDC* | 466 | 142-150 (9) | 150-311 (162) | 1086 | 209 |
| *ZmSAMDC* | 366 | 38-46 (9) | 46-198 (153) | 1203 | 304 |
| *BjSAMDC*1 | 397 | 139-150 (12) | 150-305 (156) | 1107 | 149 |
| *BjSAMDC*2 | 411 | 129-140 (12) | 140-298 (159) | 1110 | 155 |
| *DecSAMDC* | 538 | 216-224 (9) | 224-376 (153) | 1110 | 246 |
| *PsSAMDC* | 548 | 194-202 (9) | 202-366 (165) | 1062 | 219 |
| *VfSAMDC* | 562 | 210-218 (9) | 218-382 (165) | 1062 | 171 |
| *SoSAMDC* | 6 | none | none | 1092 | 81 |
| *DicSAMDC*1 | 472 | 144-152 (9) | 152-316 (165) | 1146 | 117 |
| *Dic SAMDC2* | 502 | 148-156 (9) | 156-314 (159) | 1134 | 193 |
| *GmSAMDC* | 556 | 213-221 (9) | 221-382 (162) | 1068 | 200 |
| *HvSAMDC* | 512 | 187-195 (9) | 195-344 (150) | 1182 | 199 |

**Table S4** Putative *cis* elements in the *AtSAMDC*1 promoter sequence [analyzed using Athena promoter analysis software, 46] showing consensus sequences, binding sites, and physiological responses of the identified elements.

| **Motif name** | **Consensus sequence** | **Binding sites** | **Physiological responses** |
| --- | --- | --- | --- |
| ABRE-like binding site motif | CACGTGTC | -433 to -426 | Dehydration, low temperature |
| ACGTABREMOTIFA2OSEM | ACGTGTC | -432 to -426 | ABA responsive expression |
| BoxII promoter motif | GGTTAA | -414 to -409 | light activation |
| CACGTGMOTIF | CACGTG | -433 to -428 | essential for beta phaseolin gene expression during embryogenesis |
| GADOWNAT | ACGTGTC | -432 to -426 | GA down regulated expression during seed germination |
| GAREAT | TTTGTTA | -282 to -276 | GA induced seed germination |
| MYB binding site promoter | CACCTACC | -230 to -223 | Flower specific motif |
| MYB1AT | TGGTTA | -415 to -410,  -644 to -639 | drought responsive element |
| MYB4 binding site motif | ACCTACC | -229 to -223 | drought, salt, cold, wounding |
| TATA-box Motif | TATAAA | -127 to -122,  -66 to -61, -384  to -379 | Transcription |
| W-box promoter motif | GGTCAA | -108 to -103,  -657 to -652 | Wounding response |
| Z-box promoter motif | ACACGTAT | -449 to -442 | light independent developmental expression |

**Table S5** Putative *cis* elements in the *AtSAMDC*2 promoter sequence [analyzed using Athena promoter analysis software, 46] showing consensus sequences, binding sites, and physiological responses of the identified elements.

| **Motif name** | **Consensus sequence** | **Binding sites** | **Physiological responses** |
| --- | --- | --- | --- |
| ABRE-like binding site motif | CACGTGTA and  TCCACGTG | -458 to -451,  -267 to -260,  -269 to -262 | dehydration, low temperature |
| ACGTABREMOTIFA2OSEM | ACGTGTC | -478 to -472,  -288 to -282,  -266 to -260 | ABA responsive expression |
| CACGTGMOTIF | CACGTG | -458 to -453,  -267 to -262 | essential for beta phaseolin gene expression during embryogenesis |
| GADOWNAT | ACGTGTC | -478 to -472,  -266 to -260 | GA down regulated expression during seed germination |
| GAREAT | TAACAAG | -665 to -659,  -621 to -615 | GA induced seed germination |
| I box promoter motif | CTTATC | -333 to -328 | light regulated expression |
| MYB1AT | TGGTTA | -729 to -724 | dehydration |
| MYB4 binding site motif | ACCAAAC | -687 to -681 | drought, salt, cold, wounding |
| RAV1-B binding site motif | CACCTG | -513 to -508 | domain for DNA binding protein |
| TATA-box Motif | TATAAA | -31 to -26 | transcription |

**Table S6** Putative *cis* elements in the *AtSAMDC*3 promoter sequence [analyzed using Athena promoter analysis software, 46] showing consensus sequences, binding sites, and physiological responses of the identified elements.

| **Motif name** | **Consensus sequence** | **Binding sites** | **Physiological responses** |
| --- | --- | --- | --- |
| AtMYB2 BS in RD22 | CTAACCA | -665 to -659 | drought and ABA |
| MYB binding site promoter | AACCTAAC  GTTTGGTT | -2655 to -2648  -238 to -231 | flower specific motif |
| SV40 core promoter motif | CAATCCAC  CTAACCAC | -503 to -496  -665 to -658 | enhancer |
| MYB1AT | TAACCA  TGGTTT  TGGTTA  TGGTTT | 664 to -659  -715 to -710  -1485 to -1480  -2376 to -2371 | drought responsive element |
| GAREAT | TTTGTTATTTGTA | -1195 to -1189  -2271 to -2265 | GA induced seed germination |
| TATA-box Motif | TATAAA  TATAAA  TTTATA  TTTATA  TTTATA  TTTATA  TTTATA | -2343 to -2338  -484 to -479  -408 to-403  -555 to -550  -1336 to -1331  -1840 to -1835  -2692 to -2687 | transcription |
| DREB1A/CBF3 | GCCGACTT | -2970 to -2963 | drought response |
| MYB2AT | TAACTG  TAACTG  CAGTTA | -2894 to -2889  -2310 to -2305  -1473 to -1468 | drought responsive element |
| ATHB2 binding site motif | TAATTATTA | -1905 to -1897 | environmental stresses |
| TELO-box promoter motif | AAACCCTAA  TTAGGGTTT  TTAGGGTTT | -1328 to -1320  -127 to -119  -161 to-153 | root specific expression |
| CARGCW8GAT | CAATAAAATGCTA  TTATATGCTAATTT  AAGCTTTTTATTG  CTTTTTATTGCTAA  TTTAAGCTATTAT  ATGCAATAAAATG | -2571 to -2562  -975 to -966  -877 to -868  -25 to -16  -25 to -16  -877 to -868  -975 to -966  -2571 to -2562 | ABA- mediated inhibition of seed germination |
| DRE core motif | GCCGACGCCGAC | -2970 to -2965  -1627 to -1622 | drought, salt and freezing |

**Table S6** (continued)

| **Motif name** | **Consensus sequence** | **Binding sites** | **Physiological responses** |
| --- | --- | --- | --- |
| MYB4 binding site motif | ACCTAAC  AACAAAC  AACAAAC  AACAAAC  GTTTGTT  GTTTGGT  GTTTGTT | -2654 to -2648  -1722 to -1716  -1105 to -1099  -729 to -723  -156 to -150  -238 to -232  -281 to -275 | drought, salt, cold, wounding |
| CCA1 motif1 BS in CAB1 | TAGATTGTTT | -563 to -554 | phytochrome regulation |
| W-box promoter motif | TTGACTAGTCAA | -206 to -201  -2739 to -2734 | wounding response |
| CCA1 binding site motif | AGATTGTTAGATTGTT | -243 to -236  -562 to -555 | phytochrome regulation |
| L1-box promoter motif | TAAATGTA | -588 to -581 | layer specific gene expression |
| I-box promoter motif | GATAAG | -1408 to -1403 | light regulated expression |
| GCC-box promoter motif | GCCGCC | -1630 to -1625 | dehydration and low temperature |
| RY-repeat promoter motif | CATGCATG  CATGCATG | -572 to -565  -572 to -565 | seed protein related |
| Hexamer promoter motif | CCGTCGCGACGG | -2975 to -2970  -2888 to -2883 | histone protein related |
| CACGTG-motif | CACGTG | -1206 to -1201 | essential for beta phaseolin gene expression during embryogenesis |

**Table S7** Putative *cis* elements in the *AtSAMDC*4 promoter sequence [analyzed using Athena promoter analysis software, 46] showing consensus sequences, binding sites, and physiological responses of the identified elements.

| **Motif name** | **Consensus sequence** | **Binding sites** | **Physiological responses** |
| --- | --- | --- | --- |
| ARF binding site motif | GAGACA  GAGACA | -720 to -715  -843 to -838 | auxin responsive element |
| BoxII promoter motif | TTAACC  TTAACC | 182 to -177  -361 to -356 | Transcriptional activator |
| MYB1AT | AAACCA  AAACCA  TAACCA | -980 to -975  -515 to -510  -360 to -355 | drought responsive element |
| MYB1LEPR | AACTAAC | -434 to -428 | defense related gene expression |
| MYB4 binding site motif | AACTAAC | -434 to -428 | drought, salt, cold, wounding |
| RAV1-B binding site motif | CACCTG | -423 to -418 | DNA binding domain |
| T-box promoter motif | ACTTTG | -564 to -559 | transcriptional activator |
| TATA-box Motif | TATAAA  TATAAA  TTTATA | 402 to -397  -139 to -134  -684 to -679 | transcription |
| W-box promoter motif | AGTCAA | -528 to -523 | wound response |

**Table S8** Putative *cis* elements in the *AtSAMDC*5 promoter sequence [analyzed using Athena promoter analysis software, 46] showing consensus sequences, binding sites, and physiological responses of the identified elements.

| **Motif name** | **Consensus sequence** | **Binding sites** | **Physiological responses** |
| --- | --- | --- | --- |
| ARF binding site motif | TGTCTC | -531 to -526 | auxin responsive element |
| AtMYC2 BS in RD22 | CATGTG  CATGTG | -862 to -857  -1359 to -1354 |  |
| BoxII promoter motif | GGTTAA  GGTTAA  TTAACC  TTAACC | -1672 to -1667  -1354 to -1349  -382 to -377  -1352 to -1347 | transcriptional activator |
| CARGCW8GAT | CTATTTATTG  CAATTTTTTG  CTTTTATAAG  CTTTTATAAG  CAATTTTTTG  CTATTTATTG | -2222 to -2213  -780 to -771  -96 to -87  -96 to -87  **-780 to -771**  -2222 to -2213 | ABA- mediated inhibition of seed germination |
| CCA1 binding site motif | AAAAATCT | -187 to -180 | phytochrome regulation |
| E2F binding site motif | TTTCCCGC | -933 to -926 | cell cycle regulation |
| GAREAT | TAACAAA  CTTGTTA | -877 to -871  -899 to -893 | GA induced seed germination |
| I-box promoter motif | GATAAG  CTTATC  CTTATC  CTTATC | -2354 to -2349  -996 to -991  -1209 to -1204  -1383 to -1378 | light regulated expression |
| MYB binding site promoter | GTTAGGTG  GTTTGGTG | -493 to -486  -2304 to -2297 | flower specific motif |
| MYB1AT | TGGTTA  TGGTTT  TGGTTA | -1355 to -1350  -1644 to -1639  -2104 to -2099 | drought responsive element |
| MYB1LEPR | GTTAGTT | -599 to -593 | defense related gene expression |
| MYB2AT | TAACTG  TAACTG | -2359 to -2354  -2030 to -2025 | drought responsive element |
| MYB4 binding site motif | GTTAGGT  GTTAGTT  GTTTGTT  GTTTGGT | -493 to -487  -599 to -593  -1548 to -1542  -2304 to -2298 | drought, salt, cold, wounding |
| MYCATERD1 | CATGTG  CATGTG | -1359 to -1354  -862 to -857 | signal transduction |
| RAV1-B binding site motif | CAGGTG | -2205 to -2200 | DNA-binding |
| RY-repeat promoter motif | CATGCATG  CATGCATG | -1363 to -1356  -1363 to -1356 | seed protein related |

**Table S8** (continued)

| **Motif name** | **Consensus sequence** | **Binding sites** | **Physiological responses** |
| --- | --- | --- | --- |
| T-box promoter motif | ACTTTG  CAAAGT  CAAAGT | **-702 to -697**  -553 to -548  -2456 to -2451 | transcriptional activator |
| TATA-box Motif | TATAAA  TTTATA | -1251 to -1246  -94 to -89 | transcription |
| W-box promoter motif | AGTCAA | -1614 to -1609 | wound response |

**
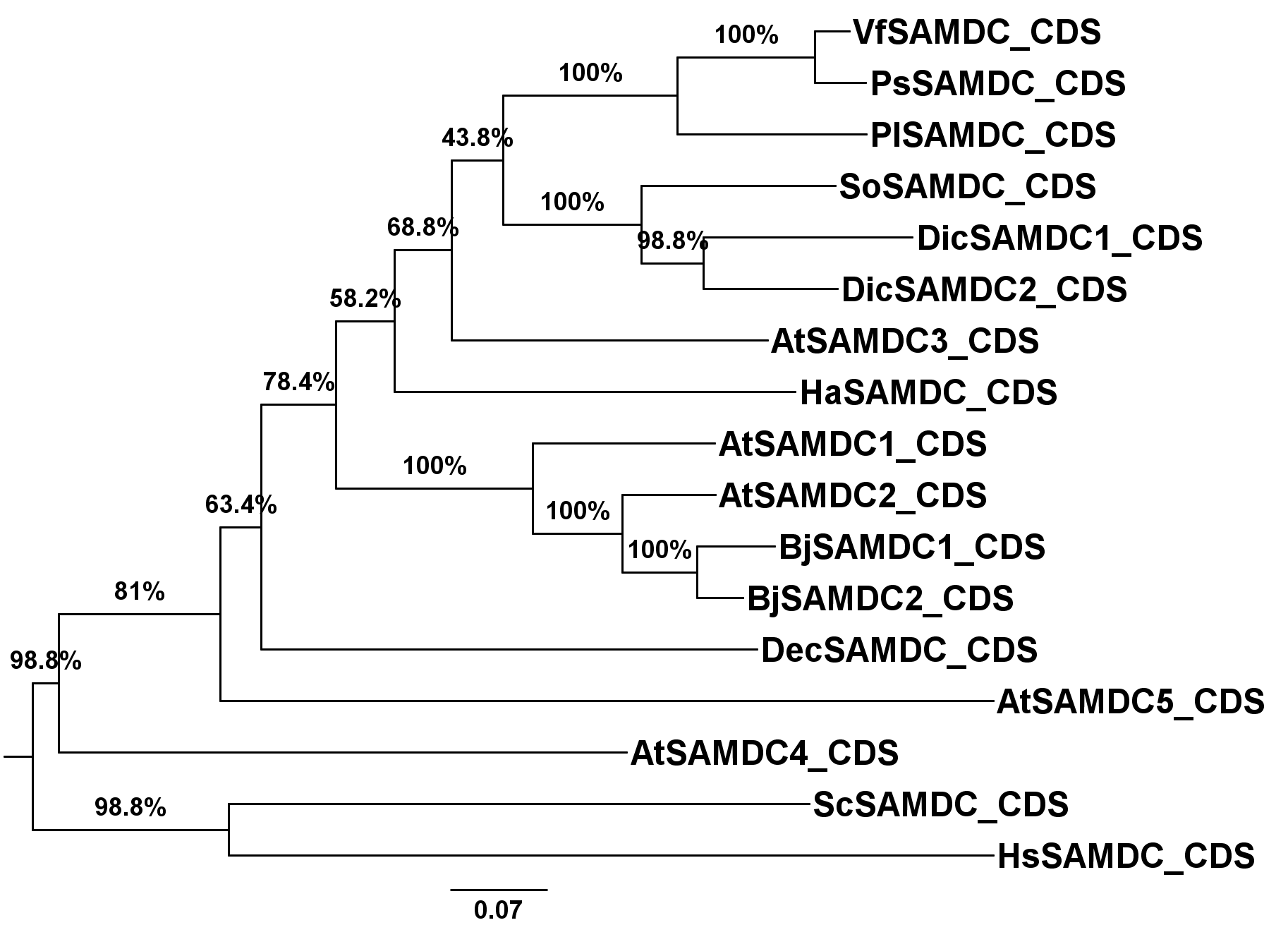
**

**Fig. S1** Phylogenetic analyses of the CDSs of Arabidopsis *SAMDC* orthologs.

**
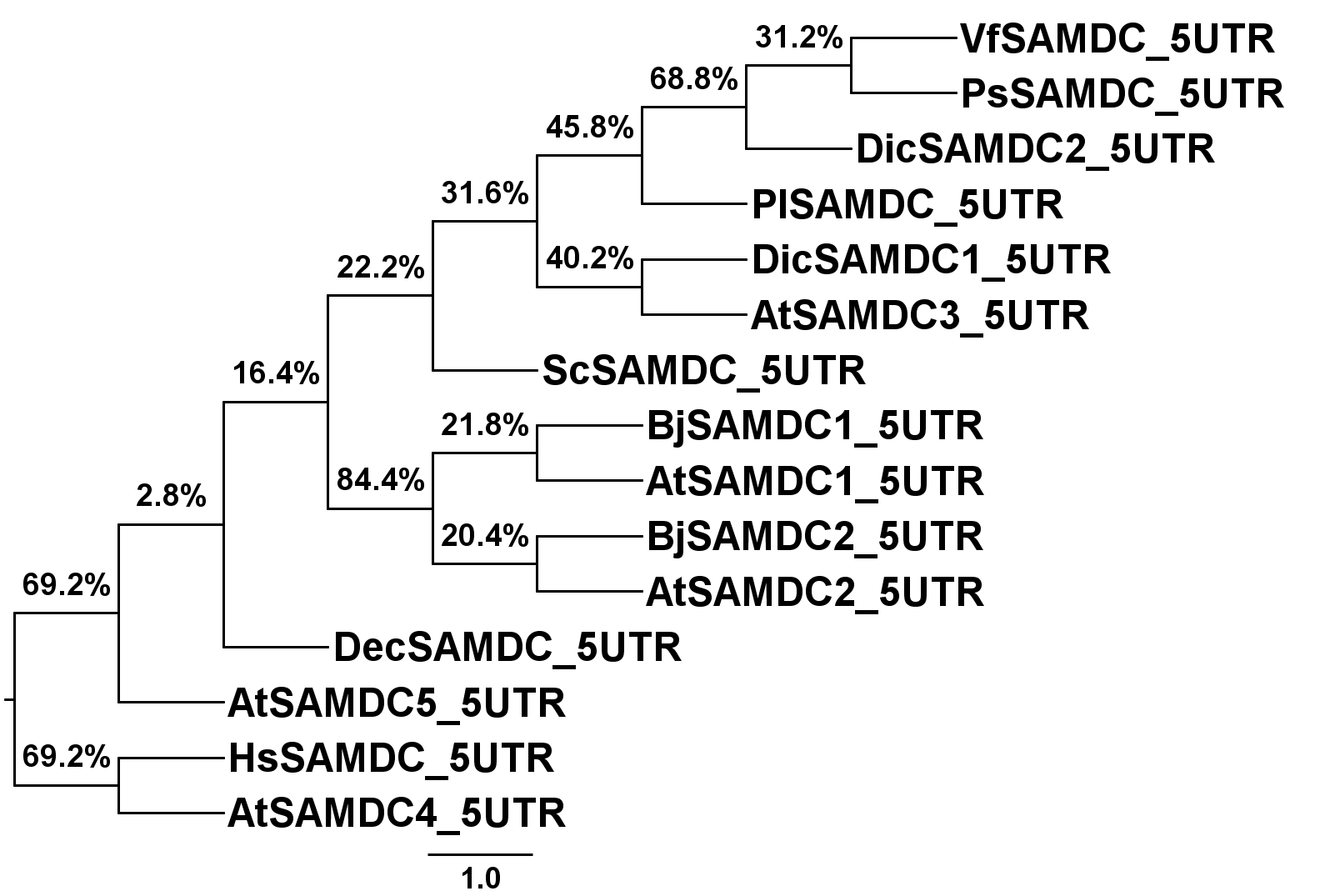
**

**Fig. S2** Phylogenetic analyses of the 5’UTRs of Arabidopsis *SAMDC* orthologs.


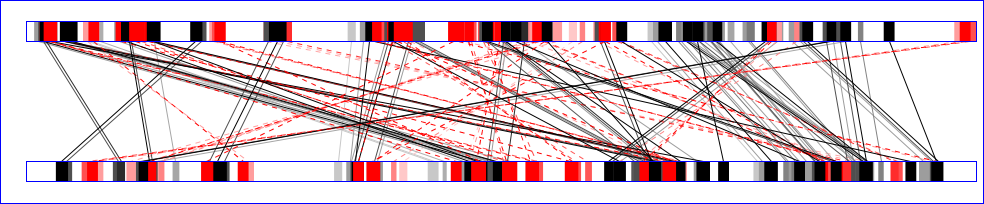

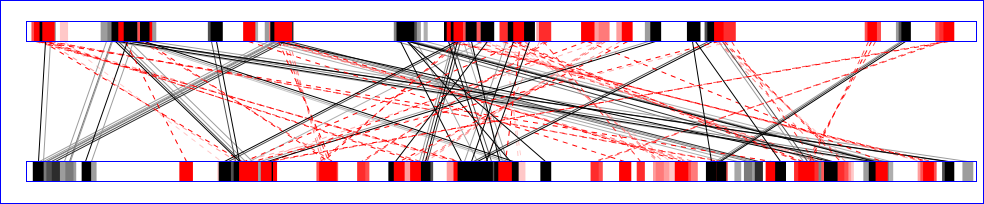

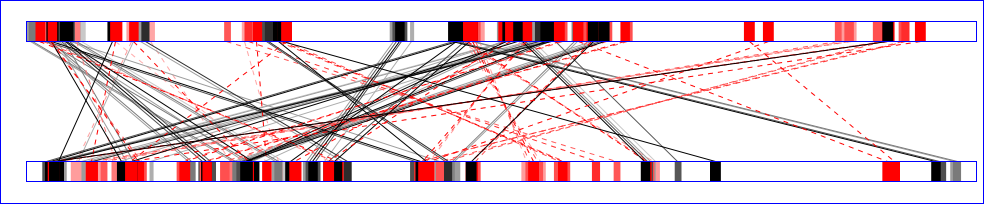

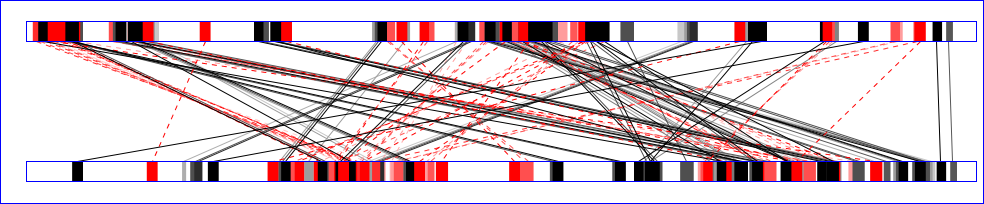

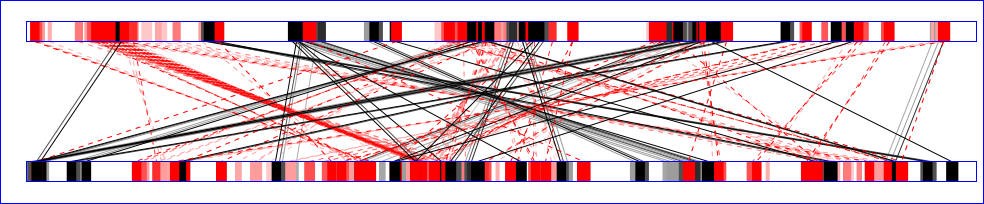

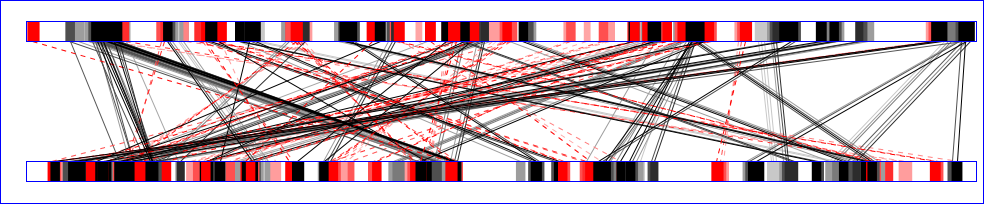

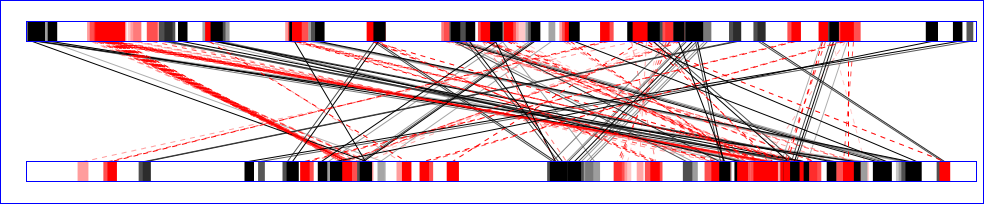

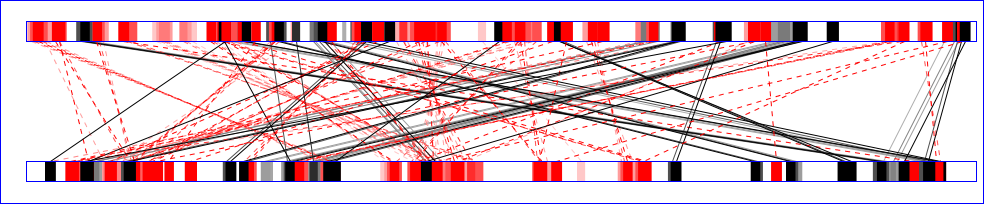

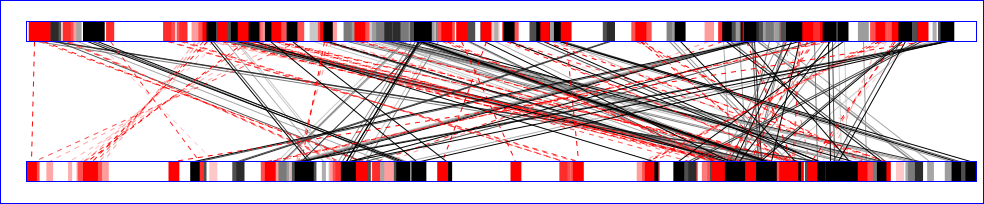

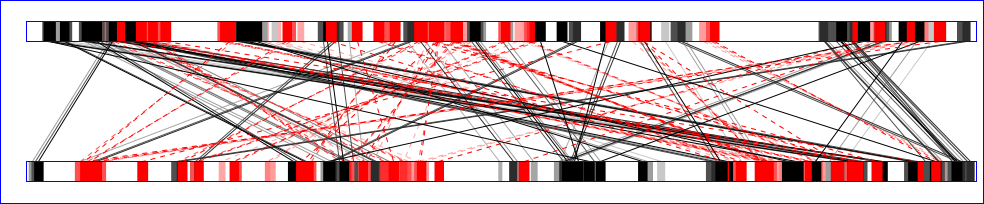


**(a)**

***SAMDC1* vs *SAMDC2***

**(b)**

***SAMDC1* vs *SAMDC3***

**(c)**

***SAMDC1* vs *SAMDC4***

**(e)**

***SAMDC2* vs *SAMDC3***

**(g)**

***SAMDC2* vs *SAMDC5***

**(i)**

***SAMDC3* vs *SAMDC5***

**(d)**

***SAMDC1* vs *SAMDC5***

**(f)**

***SAMDC2* vs *SAMDC4***

**(h)**

***SAMDC3* vs *SAMDC4***

**(j)**

***SAMDC4* vs *SAMDC5***

**Fig. S3** Comparison of the upstream (700 bp) promoter regions between different *SAMDC* genes of Arabidopsis using GATA promoter comparison software [41]. Similar regions are connected by solid black lines and dotted red lines connect matched regions in reversed orientation.

**Fig. S4** Transient expression of different constructs of *AtSAMDC* genes in poplar cells. The data represented here are from 3-6 independent shootings and expressed as % blue cells as compared to the control plasmid pCAM-*35S*::*GUS*.


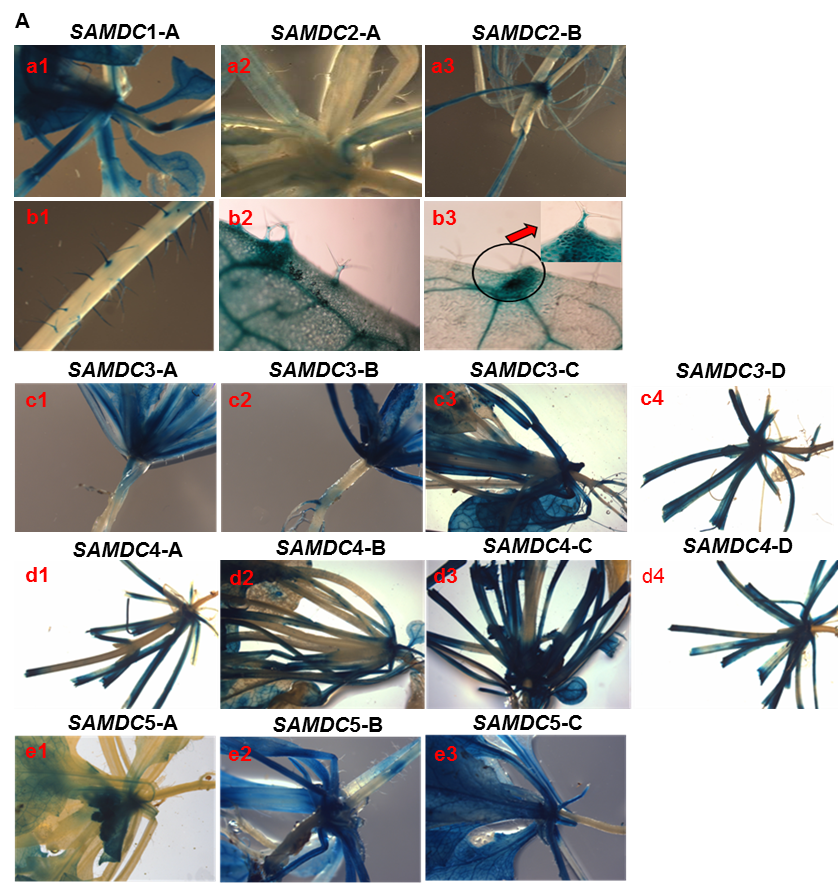


**Fig. S5** Activity of GUS in rosette junctions of 5-week old *AtSAMDC* transgenic plants. Stem and leaf trichomes of *AtSAMDC*1-A plants (b1), leaf hydathodes and trichomes of *AtSAMDC*2-A (b2, b3) plants. Similar GUS activity was observed in the *SAMDC*2-B plants.


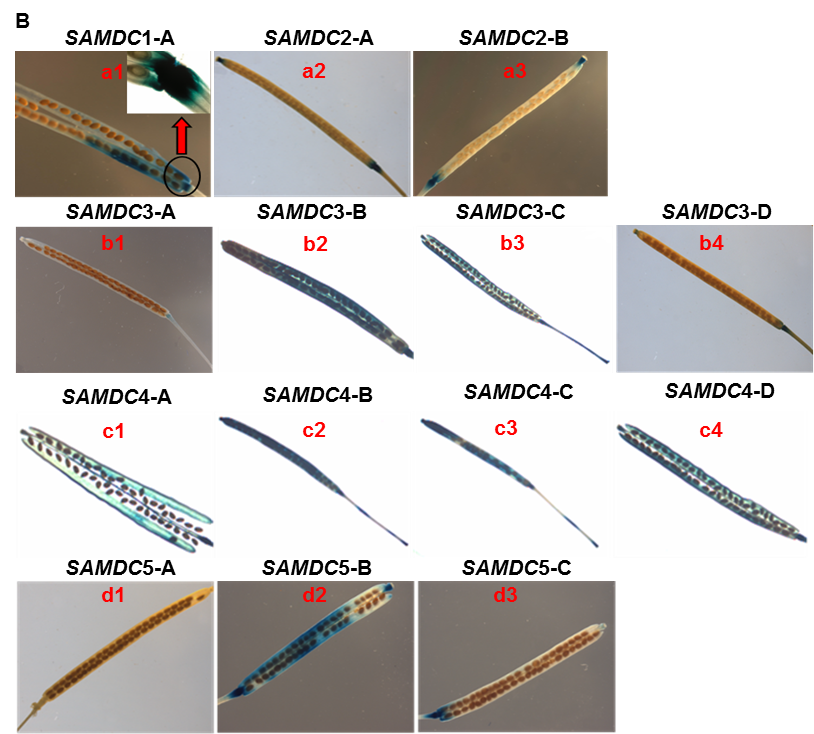


**Fig. S6** Activity of GUS in the siliques of 5-week old *AtSAMDC* transgenic plants.
